# Healthy Dietary Patterns and Risk of Major Eye Diseases: Evidence From Nationally Population‐Based Data and Bibliometric Analysis

**DOI:** 10.1002/fsn3.71528

**Published:** 2026-03-03

**Authors:** Kunhong Xiao, Jiajia Gao, Yiyan Zhang, Ruiye Chen, Yuqing Wang, Chufeng Gu, Jiahao Liu, Yan Huang, Li Li

**Affiliations:** ^1^ Shengli Clinical Medical College of Fujian Medical University, Fujian Provincial Hospital, Fuzhou University Affiliated Provincial Hospital Fuzhou China; ^2^ Department of Ophthalmology and Optometry Fujian Medical University Fuzhou China; ^3^ Centre for Eye Research Australia Royal Victorian Eye and Ear Hospital East Melbourne Australia; ^4^ Department of Surgery (Ophthalmology) The University of Melbourne Melbourne Australia; ^5^ School of Basic Medical Sciences Fujian Medical University Fuzhou China

**Keywords:** age‐related macular degeneration, bibliometric, cataract, healthy dietary patterns, NHANES, retinopathy

## Abstract

The associations between dietary patterns and major eye diseases remain incompletely understood. This study conducted a cross‐sectional analysis of 4241 participants from the 2005–2008 National Health and Nutrition Examination Survey. Eye diseases were assessed using fundus photography and self‐reported information, while dietary patterns were evaluated based on two 24‐h dietary recalls. Survey‐weighted logistic regression and restricted cubic spline models were applied with adjustment for complex sampling design and relevant covariates, alongside subgroup and dietary component–specific analyses. In fully adjusted models, higher HEI‐2020 scores were associated with lower odds of retinopathy and composite eye disease outcomes with or without cataract surgery. Similarly, greater adherence to the DASH was inversely associated with retinopathy and composite eye disease outcomes. Mediterranean diet adherence was also associated with reduced odds of retinopathy, and a non‐linear association was observed between Mediterranean diet adherence and cataract risk. Subgroup analyses indicated effect modification by age, body mass index, alcohol consumption, and hyperlipidemia status. Whole grains, total vegetables, and nuts emerged as protective dietary components, whereas refined grains were identified as a potential risk factor. In conclusion, higher adherence to HEI‐2020, DASH, and Mediterranean dietary patterns was associated with a lower prevalence of selected major eye diseases. These findings reflect associations rather than causation, and prospective studies and randomized trials are needed to clarify temporal and causal relationships.

## Graphical abstract

This study found that higher adherence to HEI‐2020, DASH, and Mediterranean dietary patterns was associated with a lower prevalence of selected major eye diseases.

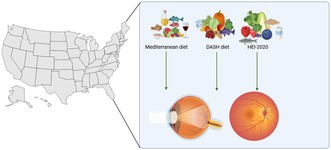

## 1 Introduction

Major eye diseases such as cataract, age‐related macular degeneration (AMD), glaucoma, and diabetic retinopathy represent leading causes of vision impairment and blindness (GBD 2019 Blindness and Vision Impairment Collaborators, and Vision Loss Expert Group of the Global Burden of Disease Study 2021). Recent Global Burden of Disease estimates indicate that more than 1 billion people live with vision impairment or blindness globally, with cataract remaining the leading cause of avoidable blindness (GBD 2019 Blindness and Vision Impairment Collaborators, and Vision Loss Expert Group of the Global Burden of Disease Study 2021). Globally, glaucoma affects 76 million people (Tham et al. 2014), AMD affects 196 million (Wong et al. 2014), and diabetic retinopathy affects 93 million individuals (Yau et al. 2012). Due to population growth and increased life expectancy, the number of individuals affected by major eye diseases has risen and is projected to increase substantially in the coming years (Kelly et al. 2020). These conditions are closely associated with reduced quality of life (Assi et al. 2021), increased national economic burden (Marques et al. 2022), and higher all‐cause mortality risk (e.g., cardiovascular disease and diabetes) (Quiruz et al. 2024) and have thus become a significant public health concern.

Dietary factors have long been recognized as critical, modifiable determinants of chronic disease (Marín‐Hinojosa et al. 2021). A growing body of research has demonstrated that adherence to healthy dietary patterns characterized by appropriate intake of fruits, vegetables, whole grains, and lean proteins is associated with reduced risk of cardiovascular disease, type 2 diabetes, and certain cancers (Shang et al. 2023). Consequently, dietary guidelines have shifted from a focus on individual nutrients toward overall dietary patterns (Reidlinger et al. 2015; English et al. 2021). Commonly used indices such as the Healthy Eating Index (HEI) (Shams‐White et al. 2023), the Mediterranean Diet (MED) score (Sofi et al. 2008), the Dietary Approaches to Stop Hypertension (DASH) score (Rahimi et al. 2020), and the Dietary Inflammatory Index (DII) (Shivappa et al. 2017) provide comprehensive evaluations of diet quality from different perspectives, including nutrient adequacy, cultural dietary habits, and inflammatory potential.

Existing literature has established links between diet and various systemic conditions, and a growing body of work has also examined dietary patterns in relation to eye health (Merle et al. 2019; Wu et al. 2023; Díaz‐López et al. 2015; Zhang, Zhou, et al. 2024). Some studies have also suggested that specific nutrients such as antioxidants (Agrón et al. 2021), omega‐3 fatty acids (Zhang et al. 2020), and carotenoids (Wu et al. 2015) may influence ocular health. However, evidence regarding the impact of holistic dietary patterns on the risk of multiple, distinct eye diseases is still limited.

National Health and Nutrition Examination Survey (NHANES) offers an ideal setting to address this question because it integrates: (i) standardized two‐day 24‐h dietary recalls that allow the calculation of multiple dietary pattern scores; (ii) a nationally representative sampling design with appropriate survey weights; and (iii) objectively graded retinal outcomes (retinopathy, AMD, and glaucoma) from fundus photography, complemented by self‐reported cataract surgery. Accordingly, this study examined the cross‐sectional associations between four widely used dietary pattern scores (HEI‐2020, MED, DASH, and DII) and the prevalence of cataract, AMD, glaucoma, and diabetic retinopathy among U.S. adults in NHANES 2005–2008.

## 2 Materials and Methods

### 2.1 Study Population and Data Resource

This study drew upon participants from the NHANES recruited between 2005 and 2008. Detailed information on the survey has been described elsewhere (https://wwwn.cdc.gov/nchs/nhanes/Default.aspx). The NHANES protocol was approved by the NCHS Research Ethics Review Board, and the survey was conducted in accordance with the Declaration of Helsinki. Written informed consent was obtained from all participants prior to participation.

**FIGURE 1:**
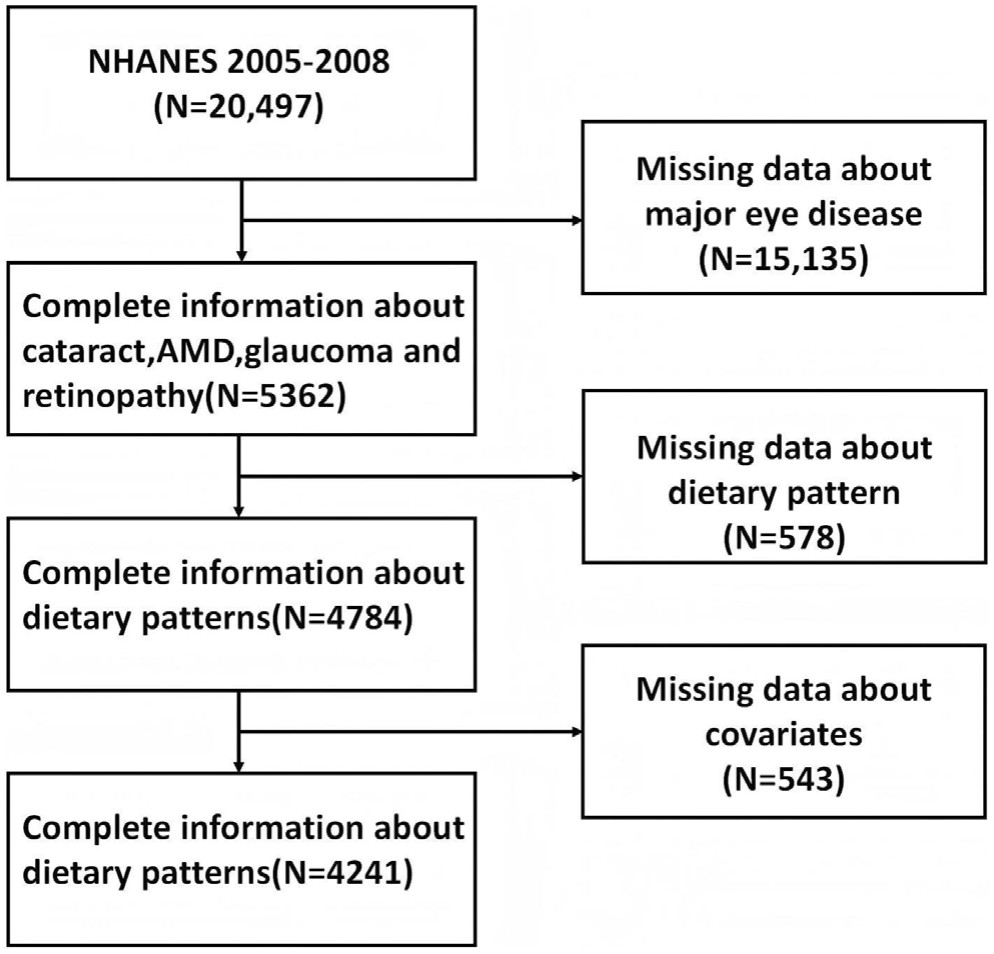
Flowchart of participant selection from NHANES 2005–2008.

Of the 20,497 participants in NHANES 2005–2008, retinal fundus photography (required for grading retinopathy, AMD, and glaucoma) was collected only among eligible participants aged ≥ 40 years who attended the Mobile Examination Center (MEC), and image‐based outcomes were additionally dependent on examination participation and photograph gradability. Therefore, participants (*n* = 15,315) who were ineligible for fundus photography by design (e.g., aged < 40 years), did not attend the MEC examination, or had ungradable/missing fundus photographs were not included in analyses of objectively assessed eye diseases. An additional 578 participants were excluded due to missing dietary pattern data, and 543 were excluded due to missing covariate data. The final analytic sample included 4241 participants with complete information (Figure 1).

### 2.2 Assessment of Major Eye Diseases

The assessment of major eye diseases was conducted following previously established methodologies (Zhu et al. 2020; Li et al. 2023). Briefly, self‐reported cataract surgery was determined based on the response to the question, “Have you ever had cataract surgery?” Given the widespread access to cataract surgery in the United States and the relatively low clinical threshold for undergoing the procedure, self‐reported cataract surgery serves as a proxy for clinically significant cataract. It is worth noting that this definition does not capture untreated or undiagnosed cataract, and the likelihood of undergoing surgery may vary according to healthcare access, insurance coverage, socioeconomic status, and care‐seeking behavior. Accordingly, cataract‐related findings should be interpreted as pertaining to surgically treated cataract rather than the full spectrum of cataract.

Early AMD was defined by the presence of soft drusen larger than 500 μm or pigmentary abnormalities, based on a modified version of the Wisconsin Age‐Related Maculopathy Grading System. Late AMD was defined by the presence of geographic atrophy, choroidal neovascularization, and/or subretinal fibrosis.

Participants with a cup‐to‐disc ratio (CDR) less than 0.6 in both eyes were classified as not having glaucoma. For those with CDR ≥ 0.6, fundus photographs were reviewed and re‐graded by glaucoma specialists. A diagnosis of glaucoma was made if a consensus grading of “probable” or “definite” was reached.

The severity of retinopathy was assessed using a modified Airlie House classification based on the Early Treatment Diabetic Retinopathy Study (ETDRS) grading scale. Participants were categorized into two groups: no retinopathy (levels 10–13) and retinopathy (levels 14–51).

The presence of any of the following conditions—cataract surgery, diabetic retinopathy, AMD, or glaucoma—was defined as “composite outcome including cataract surgery.” The presence of retinopathy, AMD, or glaucoma (excluding self‐reported cataract surgery) was defined as “composite outcome excluding cataract surgery.”

### 2.3 Dietary Pattern Assessment

Dietary pattern scores were derived from two 24‐h dietary recalls (Day 1 and Day 2). When both recalls were available, we calculated each dietary index using the two‐day combined value (i.e., the mean across Day 1 and Day 2 intakes/scores), as implemented in the dietaryindex NHANES functions. Dietary indices were calculated following a standardized algorithm (https://github.com/jamesjiadazhan/dietaryindex) in two steps: (GBD 2019 Blindness and Vision Impairment Collaborators, and Vision Loss Expert Group of the Global Burden of Disease Study 2021) estimating the intake of each food and nutrient category for each participant, and (Tham et al. 2014) calculating individual dietary index scores based on the derived intake values. Four dietary patterns were assessed in this study (Table S1):

HEI‐2020 evaluates diet quality based on adherence to the 2020–2025 Dietary Guidelines for Americans. It comprises 13 components, including 9 adequacy components (e.g., total fruits, whole fruits, total vegetables, whole grains) and 4 moderation components (e.g., added sugars, saturated fats, sodium). Each component is scored per 1000 kcal, with total scores ranging from 0 to 100. Higher scores reflect better diet quality (Shams‐White et al. 2023).

MED assesses adherence to the traditional Mediterranean dietary pattern. It includes 9 components: high intakes of vegetables, legumes, fruits and nuts, whole grains, fish, and a high ratio of monounsaturated to saturated fat; moderate alcohol consumption; and low intake of red and processed meats and dairy. Each component is scored as 1 if intake is above the median for beneficial foods (or below the median for harmful ones), yielding a total score from 0 to 9 (Fung et al. 2009).

DASH score reflects compliance with the DASH dietary pattern, which emphasizes fruits, vegetables, whole grains, low‐fat dairy, and nuts, while limiting sodium, red and processed meats, and sugary beverages. The score includes 8 components, each scored from 1 to 5 based on quintile intake, for a total score ranging from 8 to 40. Higher scores indicate greater adherence (Fung et al. 2008).

DII measures the inflammatory potential of an individual's diet, based on literature‐derived associations between specific dietary components and inflammatory biomarkers. Intakes of dietary factors are standardized against a global reference database and weighted according to their pro‐ or anti‐inflammatory effects. Higher DII scores indicate a more pro‐inflammatory diet, while lower scores indicate a more anti‐inflammatory dietary pattern (Shivappa et al. 2014).

### 2.4 Covariate Assessment

Potential confounding factors considered in this study were based on previous epidemiological research linking ocular diseases and nutrition (Zhou et al. 2022; Xiao et al. 2024). These included sociodemographic characteristics, anthropometric measurements, lifestyle factors, and comorbidities.

Sociodemographic characteristics, collected via self‐reported questionnaires, encompassed sex, age, race, education level (Some College or AA degree; High School Grad/GED; College Graduate or above; 9–11th Grade; Less Than 9th Grade), and socioeconomic status. Anthropometric measurements involved body mass index (BMI), calculated as weight in kilograms divided by height in meters squared (kg/m^2^). Lifestyle factors, assessed through questionnaires, included alcohol consumption and smoking status. Alcohol consumption was defined according to the standard NHANES criterion (Breslow et al. 2013) (≥ 12 drinks in any 1 year) and included as a covariate in all multivariable models to control for overall drinking behavior, including analyses of the Mediterranean diet score (Shan et al. 2020). Smoking status was categorized as never smokers (< 100 cigarettes in lifetime), former smokers (≥ 100 cigarettes but quit), or current smokers. Comorbidities included hypertension, hyperlipidemia, and diabetes, identified through a combination of self‐reports, medication use, and clinical laboratory assessments from NHANES.

### 2.5 Bibliometric Methods

A complementary bibliometric study was undertaken to map research activity on dietary patterns and major eye diseases. Literature searches were performed in PubMed and the Web of Science Core Collection (WoS) on 30 June 2025. The following topic search string was applied in both databases: (“dietary pattern*” OR “diet quality” OR “dietary index” OR “diet score” OR “Healthy Eating Index” OR “Mediterranean diet” OR “Dietary Approaches to Stop Hypertension” OR “Dietary Inflammatory Index” OR “food intake” OR “nutrient”) AND (“eye disease*” OR ocular OR ophthalmic OR cataract* OR “age related macular degeneration” OR glaucoma OR retinopathy OR “diabetic retinopathy”). For PubMed, results were restricted to records indexed as Clinical Trial to capture intervention‐based evidence. All WoS document types were retained. Data processing and quantitative indicators were generated using the package bibliometrix and VOSviewer 1.6.20.

### 2.6 Statistical Analysis

In descriptive analyses, categorical variables were expressed as weighted frequencies and percentages, while continuous variables were expressed as weighted medians and interquartile ranges. The complex sampling design of NHANES was accounted for using the “survey” package to perform weighted analyses. Group comparisons were made using Student's *t*‐test or the Rao–Scott Pearson chi‐square test. Weighted logistic regression (via the svyglm function with the family set to binomial or quasibinomial) was then used to examine the association between individual dietary patterns and each eye diseases. First, unadjusted univariable regressions were performed, followed by multivariable regressions that included the aforementioned covariates, with regression coefficients exponentiated to yield odds ratios (OR) and 95% confidence intervals (CI). To explore potential nonlinear relationships, restricted cubic splines (RCS) were fitted using the “rms” package, and both overall and nonlinear effects were tested. Additionally, we conducted separate regression analyses for each component of the MED, DASH and HEI‐2020 scores and performed subgroup analyses to further explore the associations. Results were visualized using forest plots. All *p*‐values were two‐sided, with statistical significance defined as *p* < 0.05. All analyses in this study were performed using R software (version 4.4.1).

## 3 Result

### 3.1 Characteristics of the Study Population

A total of 4241 participants were included in the study, representing an estimated 90,571,207 individuals in the U.S. population, with a mean age of 54.00 years. Among them, 2100 (47%) were male and 2141 (53%) were female. Participants with eye diseases were more likely to be older, have higher levels of glycohemoglobin, be current smokers, and have hypertension. Those with cataracts tended to have higher DII, HEI‐2020, MED, and DASH scores. Participants with AMD were more likely to have higher HEI‐2020 and DASH scores. Individuals with composite outcome including cataract surgery tended to have higher DII and HEI‐2020 scores. More detailed information is presented in Table 1 and Table S2.

**TABLE 1:** Baseline characteristics of participants with and without AMD, cataract, glaucoma and retinopathy.

Characteristic	Overall *N* ^a^ = 4241 (*n* ^b^ = 90,571,207)	Non‐AMD *N* = 3924	AMD *N* = 317	*p*	Non‐Cataract *N* = 3747	Cataract *N* = 494	*p*
**Age (years)**	54.00 (47.00, 64.00)	53.00 (46.00, 63.00)	70.00 (58.00, 79.00)	< 0.001	53.00 (46.00, 61.00)	75.00 (67.00, 80.00)	< 0.001
**Sex**				0.6			< 0.001
Female	2141 (53%)	1983 (53%)	158 (55%)		1868 (52%)	273 (64%)	
Male	2100 (47%)	1941 (47%)	159 (45%)		1879 (48%)	221 (36%)	
**Race**				< 0.001			0.001
Non‐Hispanic White	2411 (80%)	2178 (79%)	233 (89%)		2048 (79%)	363 (88%)	
Non‐Hispanic Black	802 (8.5%)	776 (8.8%)	26 (3.5%)		744 (8.8%)	58 (5.0%)	
Mexican American	633 (5.1%)	596 (5.1%)	37 (4.0%)		595 (5.3%)	38 (2.6%)	
Other Hispanic	275 (2.9%)	259 (3.0%)	16 (2.0%)		247 (3.0%)	28 (2.1%)	
Other Race	120 (3.8%)	115 (4.0%)	5 (1.6%)		113 (4.0%)	7 (2.2%)	
PIR				0.6			0.6
At or above poverty line	3633 (92%)	3363 (92%)	270 (91%)		3200 (92%)	433 (93%)	
Below poverty line	608 (8.1%)	561 (8.0%)	47 (8.7%)		547 (8.1%)	61 (7.5%)	
**Education**				0.2			< 0.001
Some College or AA degree	1111 (29%)	1033 (29%)	78 (28%)		997 (29%)	114 (27%)	
High School Grad/GED	1051 (26%)	961 (26%)	90 (28%)		923 (26%)	128 (28%)	
College Graduate or above	935 (29%)	874 (29%)	61 (24%)		844 (29%)	91 (21%)	
9‐11th Grade	631 (11%)	593 (11%)	38 (11%)		560 (10%)	71 (13%)	
Less Than 9th Grade	513 (5.9%)	463 (5.6%)	50 (9.4%)		423 (5.3%)	90 (12%)	
**BMI**	28.19 (24.75, 32.41)	28.20 (24.75, 32.43)	27.59 (24.76, 31.80)	0.3	28.25 (24.81, 32.55)	27.65 (24.15, 31.01)	0.003
**Glycohemoglobin (%)**	5.50 (5.20, 5.80)	5.50 (5.20, 5.80)	5.60 (5.40, 5.90)	0.003	5.50 (5.20, 5.80)	5.70 (5.40, 6.10)	< 0.001
**Drink status**				0.018			< 0.001
Drink	2930 (73%)	2723 (74%)	207 (66%)		2634 (74%)	296 (61%)	
Non drink	1311 (27%)	1201 (26%)	110 (34%)		1113 (26%)	198 (39%)	
**Diabetes status**				0.4			< 0.001
Non diabetes	3613 (89%)	3343 (89%)	270 (88%)		3234 (90%)	379 (79%)	
Diabetes	628 (11%)	581 (11%)	47 (12%)		513 (9.7%)	115 (21%)	
**Smoking status**				< 0.001			< 0.001
Never smoked	2003 (48%)	1864 (49%)	139 (41%)		1785 (49%)	218 (45%)	
Former smoker	1404 (32%)	1273 (31%)	131 (44%)		1179 (30%)	225 (45%)	
Current smoker	834 (20%)	787 (20%)	47 (15%)		783 (21%)	51 (11%)	
**Hypertension status**				0.007			< 0.001
Non hypertension	2318 (59%)	2184 (60%)	134 (46%)		2123 (60%)	195 (41%)	
Hypertension	1923 (41%)	1740 (40%)	183 (54%)		1624 (40%)	299 (59%)	
Hyperlipidemia status				0.8			0.3
Non hyperlipidemia	3513 (82%)	3249 (82%)	264 (81%)		3093 (82%)	420 (85%)	
Hyperlipidemia	728 (18%)	675 (18%)	53 (19%)		654 (18%)	74 (15%)	
**DII score**	2.60 (1.12, 3.95)	2.57 (1.12, 3.93)	2.86 (1.13, 4.10)	0.3	2.55 (1.09, 3.88)	3.00 (1.40, 4.46)	0.001
**HEI2020 score**	51.37 (43.70, 60.05)	51.27 (43.55, 59.88)	53.73 (45.19, 62.75)	0.006	51.08 (43.46, 59.68)	55.76 (46.44, 63.26)	< 0.001
**MED score**	3.50 (2.50, 4.50)	3.50 (2.50, 4.50)	4.00 (3.00, 4.50)	0.10	3.50 (2.50, 4.50)	4.00 (3.00, 4.50)	0.003
**DASH score**	23.00 (20.00, 27.00)	23.00 (19.50, 27.00)	24.50 (21.00, 27.00)	0.008	23.00 (19.50, 26.50)	24.50 (21.00, 27.50)	< 0.001

Characteristic	Overall *N* ^a^ = 4241 (*n* ^b^ = 90,571,207)	Non‐Glaucoma *N* = 4120	Glaucoma *N* = 121	*p*	Non‐retinopathy *N* = 3361	Retinopathy *N* = 880	*p*
**Age (years)**	54.00 (47.00, 64.00)	54.00 (47.00, 64.00)	71.00 (62.00, 79.00)	< 0.001	53.00 (46.00, 62.00)	63.00 (52.00, 74.00)	< 0.001
**Sex**				0.066			< 0.001
Female	2141 (53%)	2095 (53%)	46 (44%)		1750 (54%)	391 (46%)	
Male	2100 (47%)	2025 (47%)	75 (56%)		1611 (46%)	489 (54%)	
**Race**				0.051			0.015
Non‐Hispanic White	2411 (80%)	2346 (80%)	65 (73%)		1923 (80%)	488 (78%)	
Non‐Hispanic Black	802 (8.5%)	764 (8.3%)	38 (17%)		604 (8.0%)	198 (11%)	
Mexican American	633 (5.1%)	623 (5.1%)	10 (4.0%)		508 (5.0%)	125 (5.3%)	
Other Hispanic	275 (2.9%)	270 (3.0%)	5 (1.3%)		224 (2.9%)	51 (3.3%)	
Other Race	120 (3.8%)	117 (3.8%)	3 (4.1%)		102 (4.1%)	18 (2.4%)	
PIR				0.3			0.3
At or above poverty line	3633 (92%)	3532 (92%)	101 (89%)		2888 (92%)	745 (91%)	
Below poverty line	608 (8.1%)	588 (8.0%)	20 (11%)		473 (7.9%)	135 (9.1%)	
**Education**				0.3			< 0.001
Some College or AA degree	1111 (29%)	1077 (29%)	34 (31%)		888 (29%)	223 (28%)	
High School Grad/GED	1051 (26%)	1021 (26%)	30 (25%)		821 (25%)	230 (29%)	
College Graduate or above	935 (29%)	917 (29%)	18 (19%)		796 (30%)	139 (20%)	
9‐11th Grade	631 (11%)	610 (11%)	21 (14%)		484 (10%)	147 (13%)	
Less Than 9th Grade	513 (5.9%)	495 (5.8%)	18 (9.5%)		372 (5.2%)	141 (9.1%)	
BMI	28.19 (24.75, 32.41)	28.19 (24.74, 32.38)	28.20 (25.70, 33.08)	0.5	28.12 (24.60, 32.31)	28.58 (25.14, 32.58)	0.2
**Glycohemoglobin (%)**	5.50 (5.20, 5.80)	5.50 (5.20, 5.80)	5.70 (5.40, 6.00)	0.006	5.50 (5.20, 5.70)	5.70 (5.40, 6.20)	< 0.001
**Drink status**				0.7			< 0.001
Drink	2930 (73%)	2847 (73%)	83 (72%)		2364 (74%)	566 (67%)	
Non drink	1311 (27%)	1273 (27%)	38 (28%)		997 (26%)	314 (33%)	
**Diabetes status**				0.002			< 0.001
No diabetes	3613 (89%)	3523 (90%)	90 (76%)		2998 (92%)	615 (75%)	
Diabetes	628 (11%)	597 (10%)	31 (24%)		363 (7.9%)	265 (25%)	
**Smoking status**				< 0.001			0.007
Never smoked	2003 (48%)	1957 (49%)	46 (39%)		1612 (49%)	391 (43%)	
Former smoker	1404 (32%)	1340 (31%)	64 (54%)		1064 (31%)	340 (37%)	
Current smoker	834 (20%)	823 (20%)	11 (6.6%)		685 (20%)	149 (19%)	
**Hypertension status**				0.009			< 0.001
No hypertension	2318 (59%)	2264 (59%)	54 (39%)		1946 (61%)	372 (48%)	
Hypertension	1923 (41%)	1856 (41%)	67 (61%)		1415 (39%)	508 (52%)	
**Hyperlipidemia status**				0.011			0.11
Non hyperlipidemia	3513 (82%)	3407 (82%)	106 (92%)		2766 (82%)	747 (85%)	
Hyperlipidemia	728 (18%)	713 (18%)	15 (8.4%)		595 (18%)	133 (15%)	
DII score	2.60 (1.12, 3.95)	2.59 (1.12, 3.95)	2.83 (1.11, 3.89)	0.9	2.56 (1.09, 3.90)	2.79 (1.16, 4.15)	0.14
HEI2020 score	51.37 (43.70, 60.05)	51.37 (43.67, 60.01)	51.24 (44.27, 60.31)	0.6	51.31 (43.57, 59.94)	51.65 (44.20, 60.59)	0.4
MED score	3.50 (2.50, 4.50)	3.50 (2.50, 4.50)	3.50 (2.50, 4.50)	0.8	3.50 (2.50, 4.50)	3.50 (2.50, 4.50)	0.5
DASH score	23.00 (20.00, 27.00)	23.00 (20.00, 27.00)	23.50 (20.00, 27.50)	0.8	23.00 (20.00, 27.00)	23.00 (20.00, 26.50)	0.5

*Note:* Continuous variables were summarized using means and interquartile ranges, while categorical variables were presented as unweighted counts and weighted percentages. Comparisons between participants with and without major eye diseases were conducted using *t*‐tests for continuous variables and design‐adjusted Rao–Scott Pearson *χ* ^2^ tests for categorical variables. Bolded variables indicate statistically significant between‐group differences.

^a^ *N* not Missing (unweighted).

^b^ *n* not Missing (weighted).

### 3.2 Association Between Dietary Patterns and Major Eye Diseases Based on Weighted Logistic Regression

**FIGURE 2:**
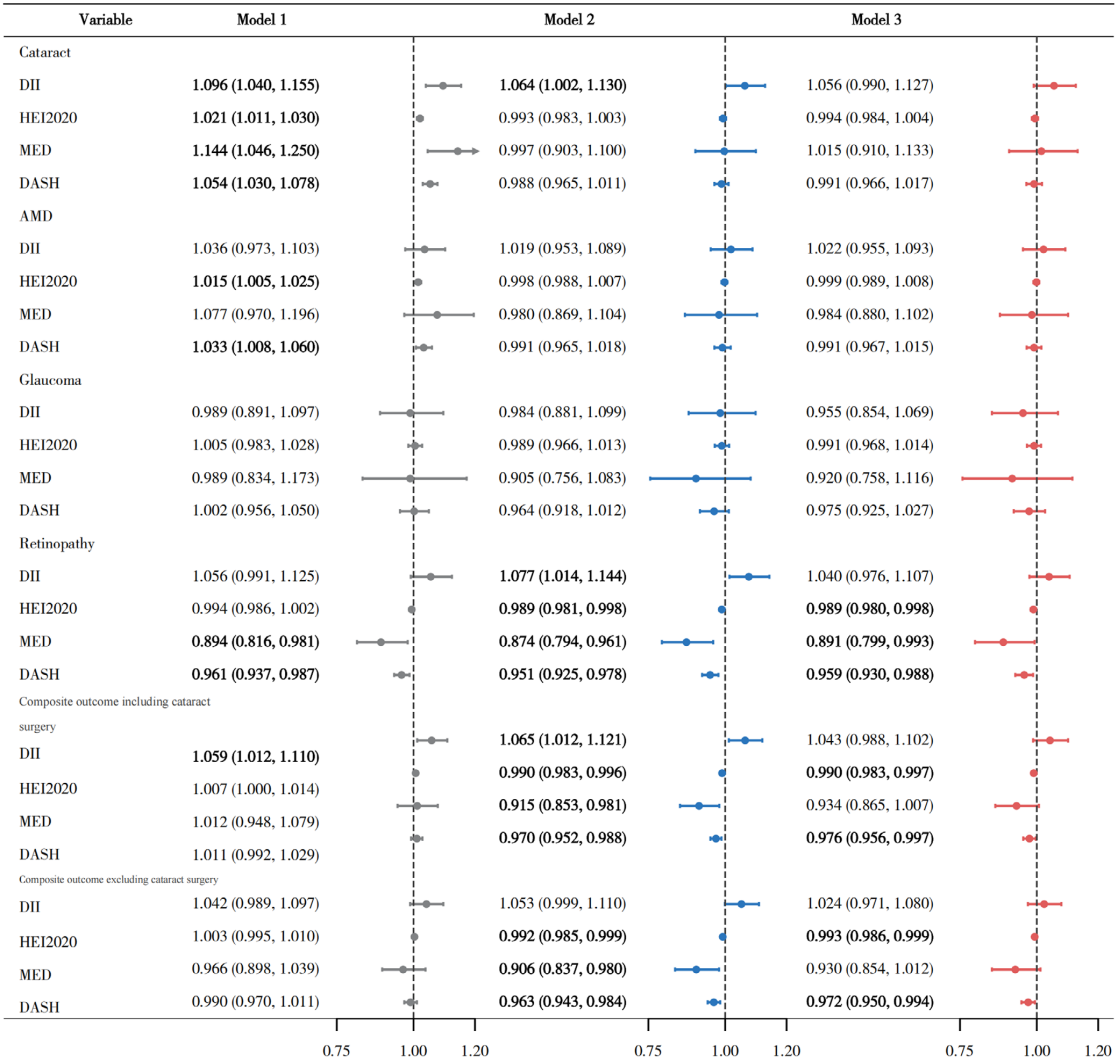
Associations between dietary patterns and major eye diseases in NHANES 2005–2008. Bolded values indicate statistically significant between‐group differences.

Weighted logistic regression models were used to examine the associations between four dietary indices and six major eye disease outcomes (Figure 2). Model 1 included only the dietary index (univariate), Model 2 adjusted for age, sex, and race, and Model 3 further adjusted for all covariates.

In Model 1, DII scores were significantly associated with cataract (OR = 1.096, 95% CI: 1.040–1.155) and composite outcome including cataract surgery (OR = 1.059, 95% CI: 1.012–1.110). Higher HEI‐2020 scores were positively associated with cataract (OR = 1.021, 95% CI: 1.011–1.030) and AMD (OR = 1.015, 95% CI: 1.005–1.025). Higher MED scores were significantly associated with increased odds of cataract (OR = 1.144, 95% CI: 1.046–1.250), and higher DASH scores were also significantly associated with cataract (OR = 1.054, 95% CI: 1.030–1.078), AMD (OR = 1.033, 95% CI: 1.008–1.060). Conversely, higher MED and DASH scores were inversely associated with retinopathy (OR = 0.894, 95% CI: 0.816–0.981; OR = 0.961, 95% CI: 0.937–0.987).

In Model 2, significant positive associations remained between DII and the odds of cataract (OR = 1.064, 95% CI: 1.002–1.130), retinopathy (OR = 1.077, 95% CI: 1.014–1.144) and composite outcome including cataract surgery (OR = 1.065, 95% CI: 1.012–1.121). Higher HEI‐2020 scores were inversely associated with retinopathy (OR = 0.989, 95% CI: 0.981–0.998), composite outcome including cataract surgery (OR = 0.990, 95% CI: 0.983–0.996) and composite outcome excluding cataract surgery (OR = 0.992, 95% CI: 0.985–0.999). MED scores were inversely associated with retinopathy (OR = 0.874, 95% CI: 0.794–0.961), composite outcome including cataract surgery (OR = 0.915, 95% CI: 0.853–0.981) and composite outcome excluding cataract surgery (OR = 0.906, 95% CI: 0.837–0.980). DASH scores were negatively associated with retinopathy (OR = 0.951, 95% CI: 0.925–0.978), composite outcome including cataract surgery (OR = 0.970, 95% CI: 0.952–0.988) and any objective eye disease (OR = 0.963, 95% CI: 0.943–0.984).

In Model 3, HEI‐2020 scores were associated with the odds of retinopathy (OR = 0.989, 95% CI: 0.980–0.998), composite outcome including cataract surgery (OR = 0.990, 95% CI: 0.983–0.997), and composite outcome excluding cataract surgery (OR = 0.993, 95% CI: 0.986–0.999). Similarly, higher DASH scores were significantly associated with lower odds of retinopathy (OR = 0.959, 95% CI: 0.930–0.988), composite outcome including cataract surgery (OR = 0.976, 95% CI: 0.956–0.997), and composite outcome excluding cataract surgery (OR = 0.972, 95% CI: 0.950–0.994). MED score remained inversely associated with the odds of retinopathy (OR = 0.891, 95% CI: 0.799–0.993). In sensitivity analyses comparing mild with moderate/severe retinopathy, no statistically significant associations were observed between retinopathy severity and any of the four dietary pattern scores (Table S3).

### 3.3 Nonlinear Associations Between Dietary Patterns and Major Eye Diseases

**FIGURE 3:**
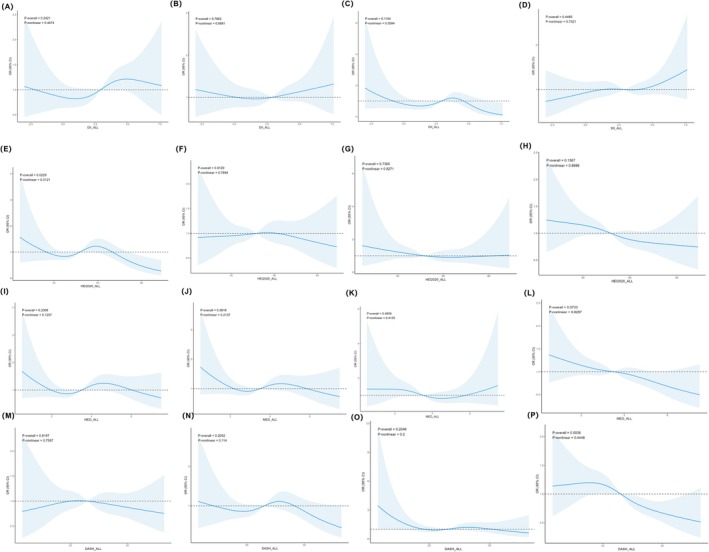
Dose response relationships between dietary indices and the risk of eye diseases in NHANES 2005–2008. (A) DII and cataract, (B) DII and AMD, (C) DII and glaucoma, (D) DII and retinopathy, (E) HEI‐2020 and cataract, (F) HEI‐2020 and AMD, (G) HEI‐2020 and glaucoma, (H) HEI‐2020 and retinopathy, (I) MED and cataract, (J) MED and AMD, (K) MED and glaucoma, (L) MED and retinopathy, (M) DASH and cataract, (N) DASH and AMD, (O) DASH and glaucoma, (P) DASH and retinopathy.

We assessed the nonlinear associations between individual dietary patterns and major eye diseases using fully adjusted, weighted RCS models with four knots (Figure 3 and Figure S1). Significant associations were observed between HEI‐2020, MED, DASH scores and the odds of composite outcome including cataract surgery (P‐overall < 0.05). In particular, DASH scores showed significant nonlinear associations with the odds of retinopathy and any objectively diagnosed eye disease (P‐overall < 0.05). Additionally, HEI‐2020 was significantly associated with cataract risk (P‐overall = 0.0229), and a significant nonlinear trend was observed (P‐nonlinear = 0.0121).

### 3.4 Subgroup Analysis

**FIGURE 4:**
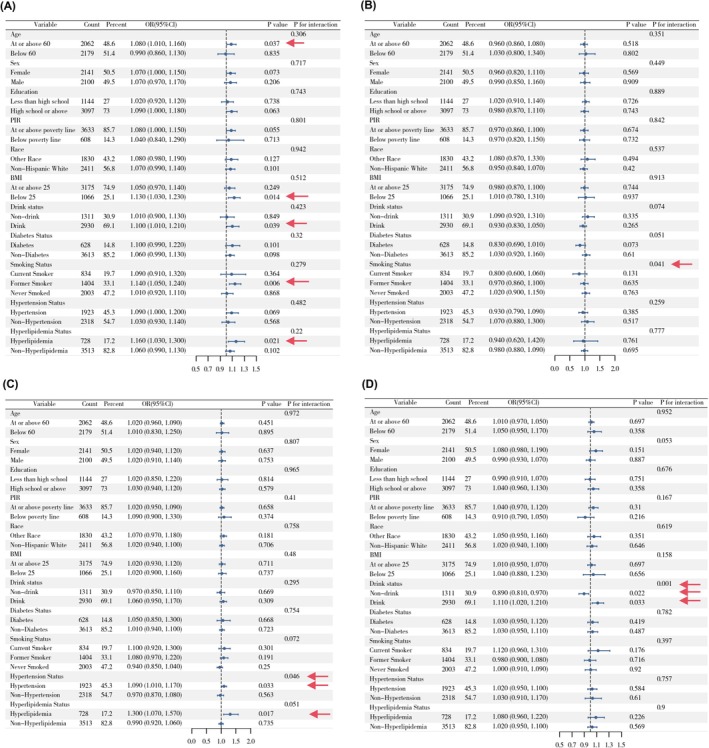
Stratified analyses of the associations between DII and eye diseases in NHANES 2005–2008. (A) DII and cataract, (B) DII and glaucoma, (C) DII and AMD, (D) DII and retinopathy.

A fully adjusted weighted logistic regression model was employed to conduct subgroup analyses across all covariates. A significant positive association between DII and cataract was observed in individuals aged ≥ 60 years, those with a BMI < 25, alcohol drinkers, and those with hyperlipidemia. Regarding the association between DII and glaucoma, smoking status demonstrated a significant interaction effect. In the hyperlipidemia subgroup, DII was positively associated with AMD, with hyperlipidemia showing a significant interaction effect. Among non‐drinkers, DII was negatively associated with retinopathy, whereas in drinkers, the association was positive; alcohol consumption had a significant interaction effect in this relationship (Figure 4). Moreover, in individuals with hyperlipidemia, DII was positively associated with both composite outcome including cataract surgery and composite outcome excluding cataract surgery, and drinking status also showed a significant interaction effect on the association between DII and composite outcome excluding cataract surgery (Figures S2 and S3).

**FIGURE 5:**
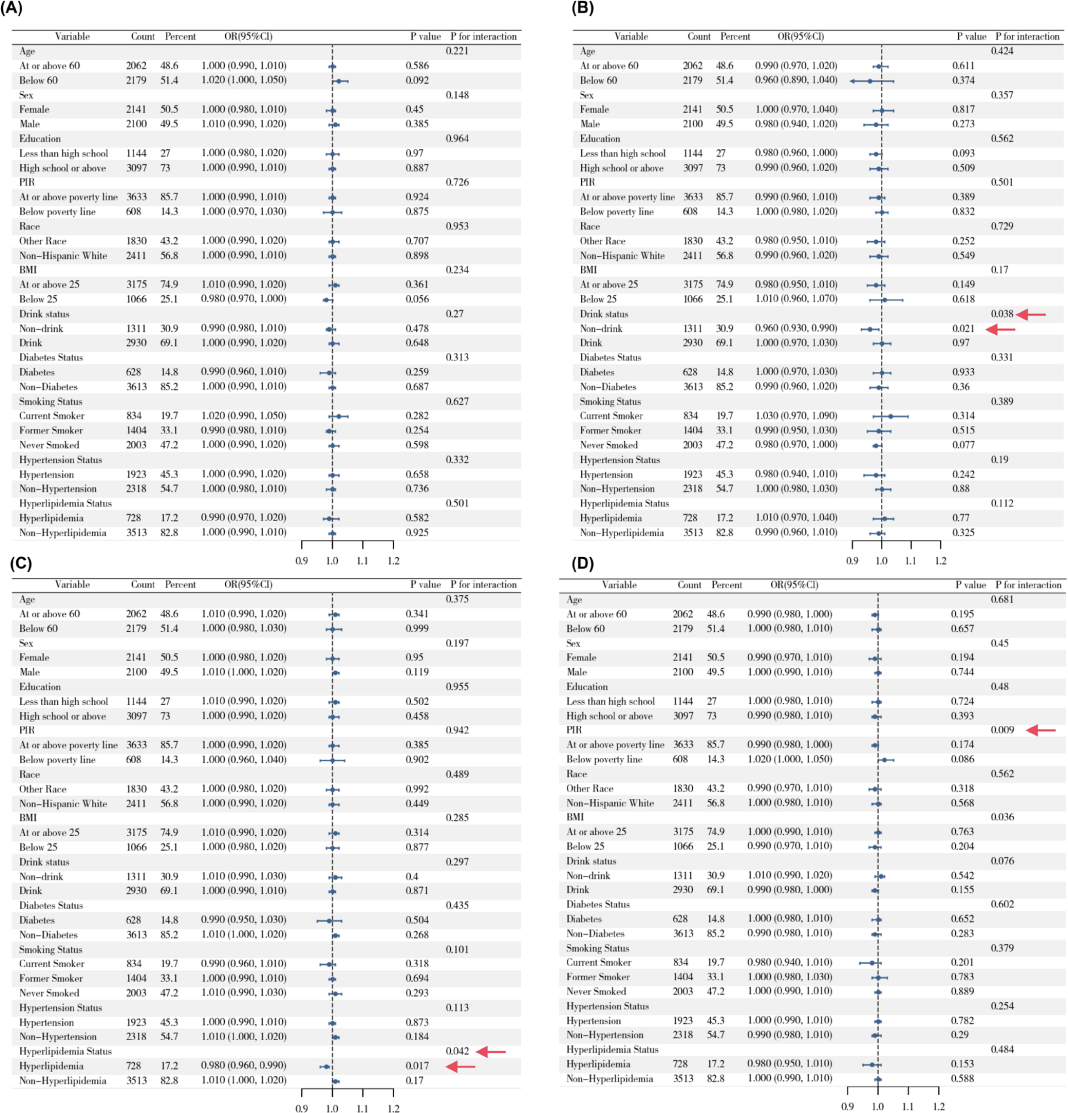
Stratified analyses of the associations between HEI‐2020 and eye diseases in NHANES 2005–2008. (A) HEI‐2020 and cataract, (B) HEI‐2020 and glaucoma, (C) HEI‐2020 and AMD, (D) HEI‐2020 and retinopathy.

A significant interaction was observed between alcohol consumption and HEI‐2020 scores in relation to the odds of glaucoma. Specifically, HEI‐2020 was negatively associated with glaucoma in the non‐drinking population. In addition, there is a significant interaction between hyperlipidemia and HEI‐2020 scores in relation to the odds of AMD, with a negative association observed in the hyperlipidemia subgroup. Moreover, both PIR and BMI showed interaction effects with the relationship between HEI‐2020 on the odds of retinopathy (Figure 5). In individuals with hyperlipidemia, HEI‐2020 was negatively associated with composite outcomes including cataract surgery and composite outcomes excluding cataract surgery (Figures S2 and S3).

**FIGURE 6:**
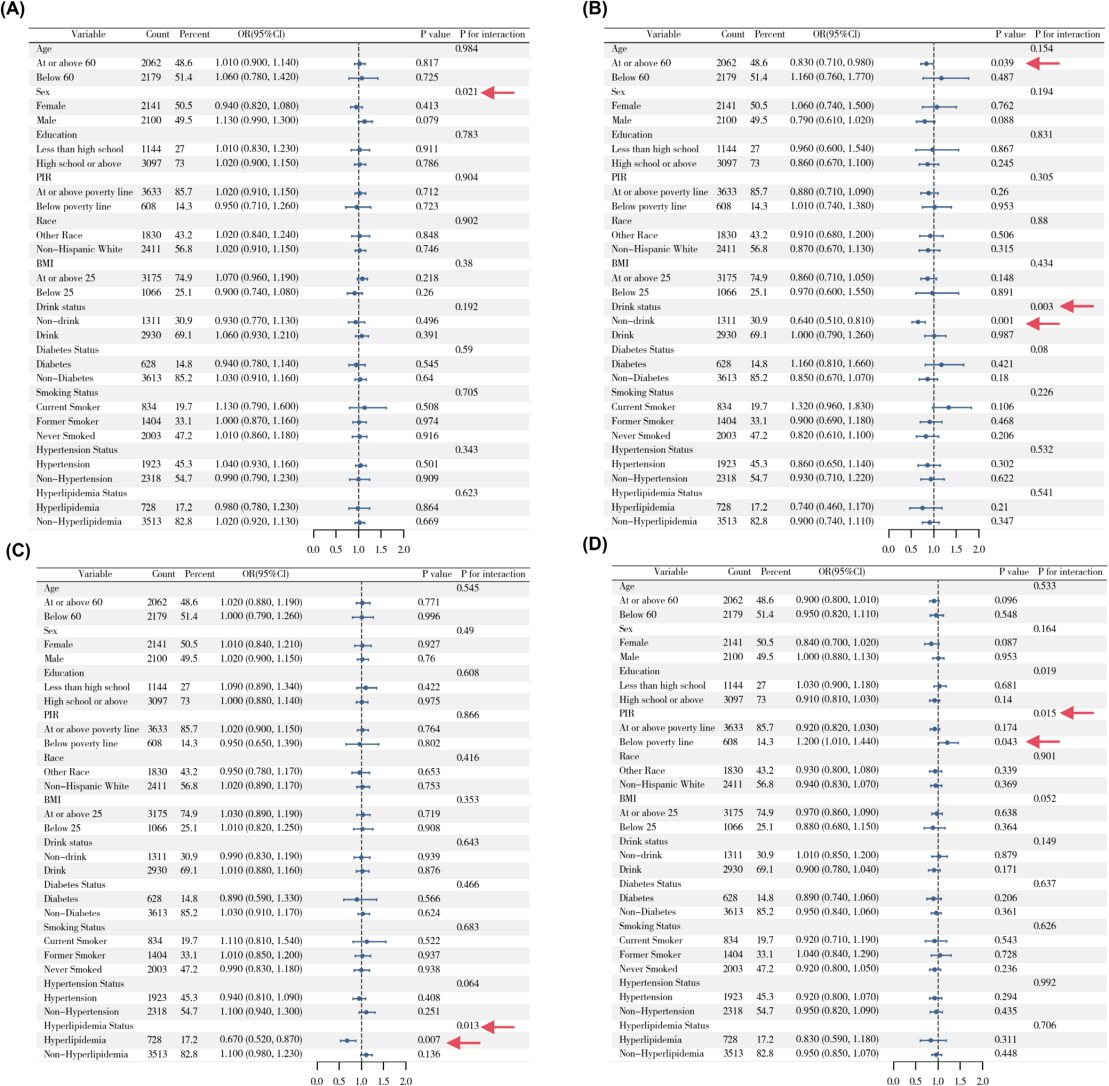
Stratified analyses of the associations between MED and eye diseases in NHANES 2005–2008. (A) MED and cataract, (B) MED and glaucoma, (C) MED and AMD, (D) MED and retinopathy.

MED was negatively associated with glaucoma in individuals aged ≥ 60 years and non‐drinkers, with drinking status showing a significant interaction effect. In the hyperlipidemia subgroup, MED was negatively associated with AMD, with hyperlipidemia acting as a significant effect modifier. Education level and PIR also exhibited interaction effects on the association between MED and retinopathy. Among individuals with hyperlipidemia, MED was significantly negatively associated with both composite outcome including cataract surgery and composite outcome excluding cataract surgery (Figure 6; Figures S2 and S3).

**FIGURE 7:**
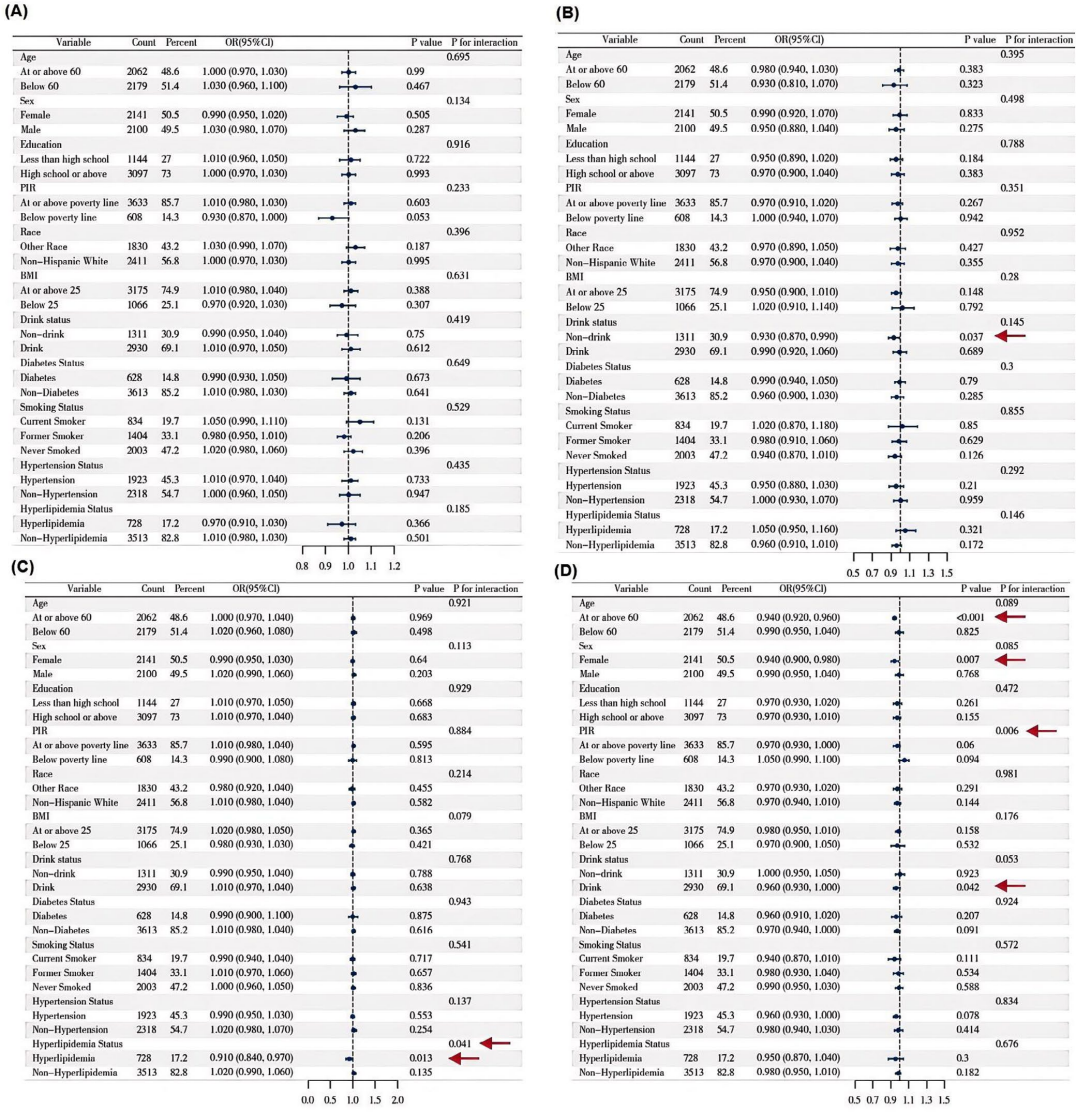
Stratified analyses of the associations between DASH and eye diseases in NHANES 2005–2008. (A) DASH and cataract, (B) DASH and glaucoma, (C) DASH and AMD, (D) DASH and retinopathy.

In non‐drinkers, DASH was negatively associated with glaucoma. Among individuals with hyperlipidemia, DASH showed a negative association with AMD, with hyperlipidemia having a significant interaction effect. Additionally, in individuals aged ≥ 60 years, females, and drinkers, DASH was negatively associated with retinopathy, with PIR demonstrating an interaction effect in this relationship. Furthermore, DASH was negatively associated with composite outcome including cataract surgery in the hyperlipidemia subgroup and with composite outcome excluding cataract surgery in individuals aged ≥ 60 years. PIR also showed a significant interaction effect in the association between DASH and composite outcome excluding cataract surgery (Figure 7 and Figure S3).

### 3.5 Associations Between Components of Dietary Patterns and Major Eye Diseases

Fully adjusted weighted logistic regression models were used to explore the associations between components of the HEI‐2020 and DASH dietary patterns and major eye diseases, as well as between components of the MED diet and retinopathy.

**FIGURE 8:**
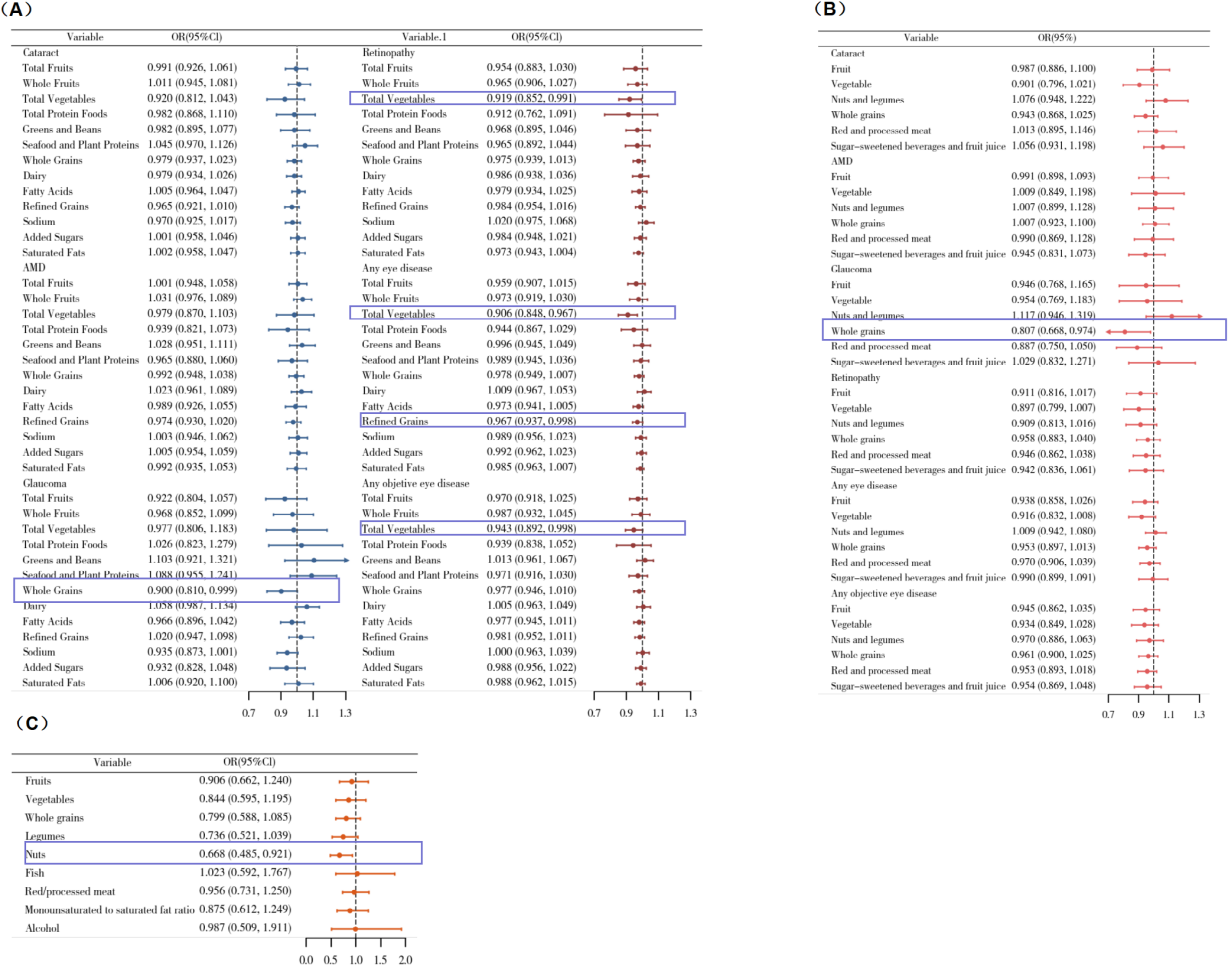
Associations between dietary pattern components and eye diseases in NHANES 2005–2008. (A) Components of HEI‐2020 in relation to cataract, AMD, glaucoma, and retinopathy. (B) Components of DASH in relation to cataract, AMD, glaucoma, and retinopathy. (C) Components of MED in relation to retinopathy.

Among the components of HEI‐2020 (Figure 8A), higher intake of whole grains was associated with a lower risk of glaucoma, while higher consumption of total vegetables was associated with reduced risks of retinopathy, composite outcome including cataract surgery, and composite outcome excluding cataract surgery. In addition, refined grains were inversely associated with the risk of composite outcome including cataract surgery.

For the DASH dietary pattern, whole grains were similarly associated with a decreased risk of glaucoma (Figure 8B). Regarding the MED dietary pattern, the nuts component was inversely associated with the risk of retinopathy (Figure 8C).

### 3.6 Bibliometric Analysis of Literature on Healthy Dietary Patterns and Eye Diseases

**FIGURE 9:**
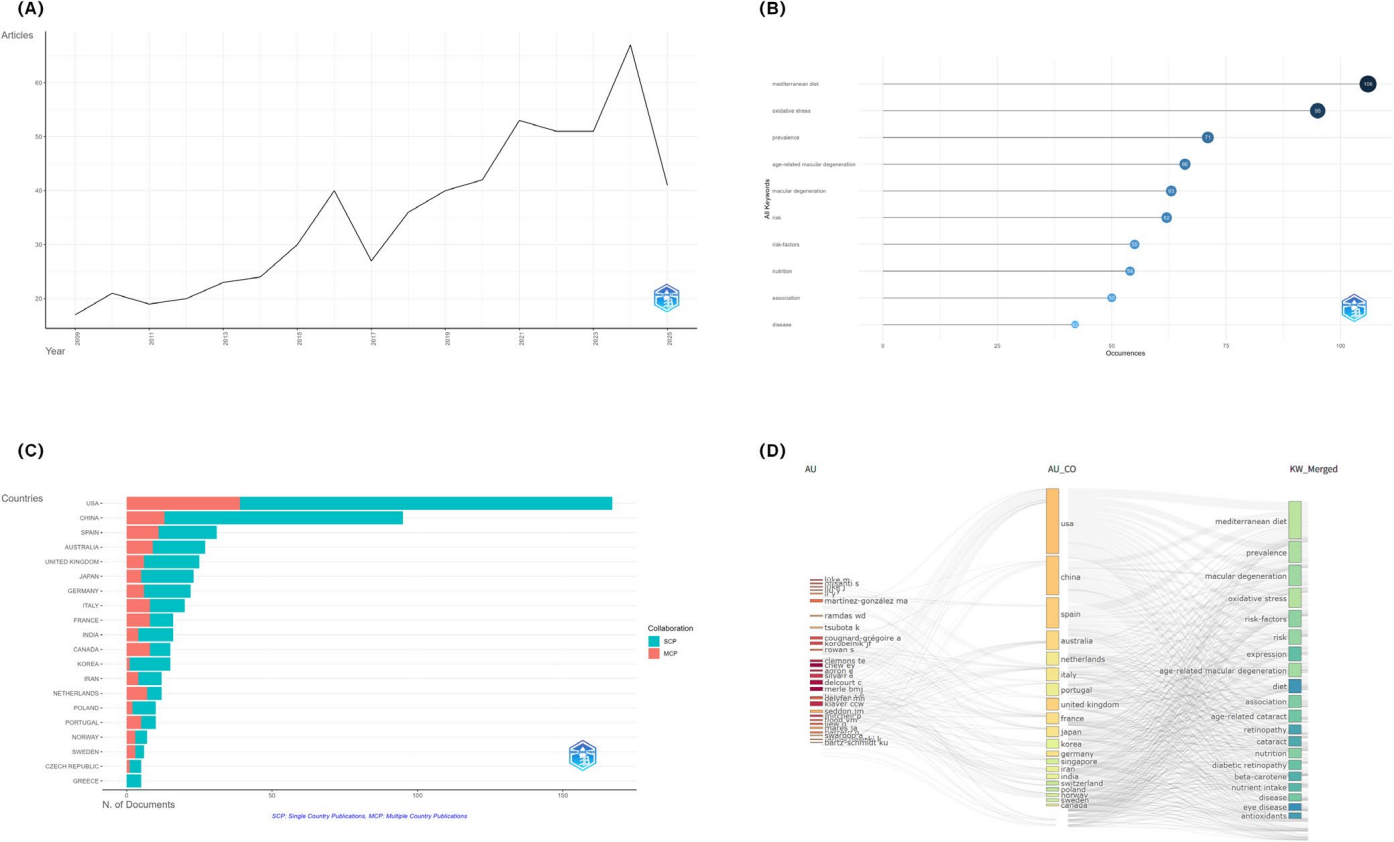
Bibliometric visualization of global research on dietary patterns and eye diseases. (A) Annual scientific production from 2009 to 2024 (B) Top 10 most frequently occurring author keywords (C) Country‐wise scientific output and international collaboration. (D) Sankey diagram linking authors (AU), affiliated countries (AU_CO), and high‐frequency keywords (KW_Merged).

Bibliometric analysis revealed a gradual increase in research on diet and eye diseases over the past decade, with a particularly notable rise after 2014 and a peak in publications in 2024 (Figure 9A). Keyword analysis indicated that “Mediterranean diet” and “oxidative stress” were among the most frequently occurring terms, suggesting a primary research focus on dietary patterns and oxidative mechanisms in age‐related eye diseases (Figure 9B). In terms of geographic distribution, the United States and China produced the highest number of publications, while European countries such as Spain and the Netherlands exhibited a higher proportion of international collaborations, reflecting more extensive cross‐border cooperation (Figure 9C). The author–country–keyword Sankey visualization further highlighted representative scholars and their research interests, such as Martínez‐González MA from Spain, who focused on the Mediterranean diet, and Tsubota K from Japan, who made significant contributions to studies on oxidative stress (Figure 9D).

**FIGURE 10:**
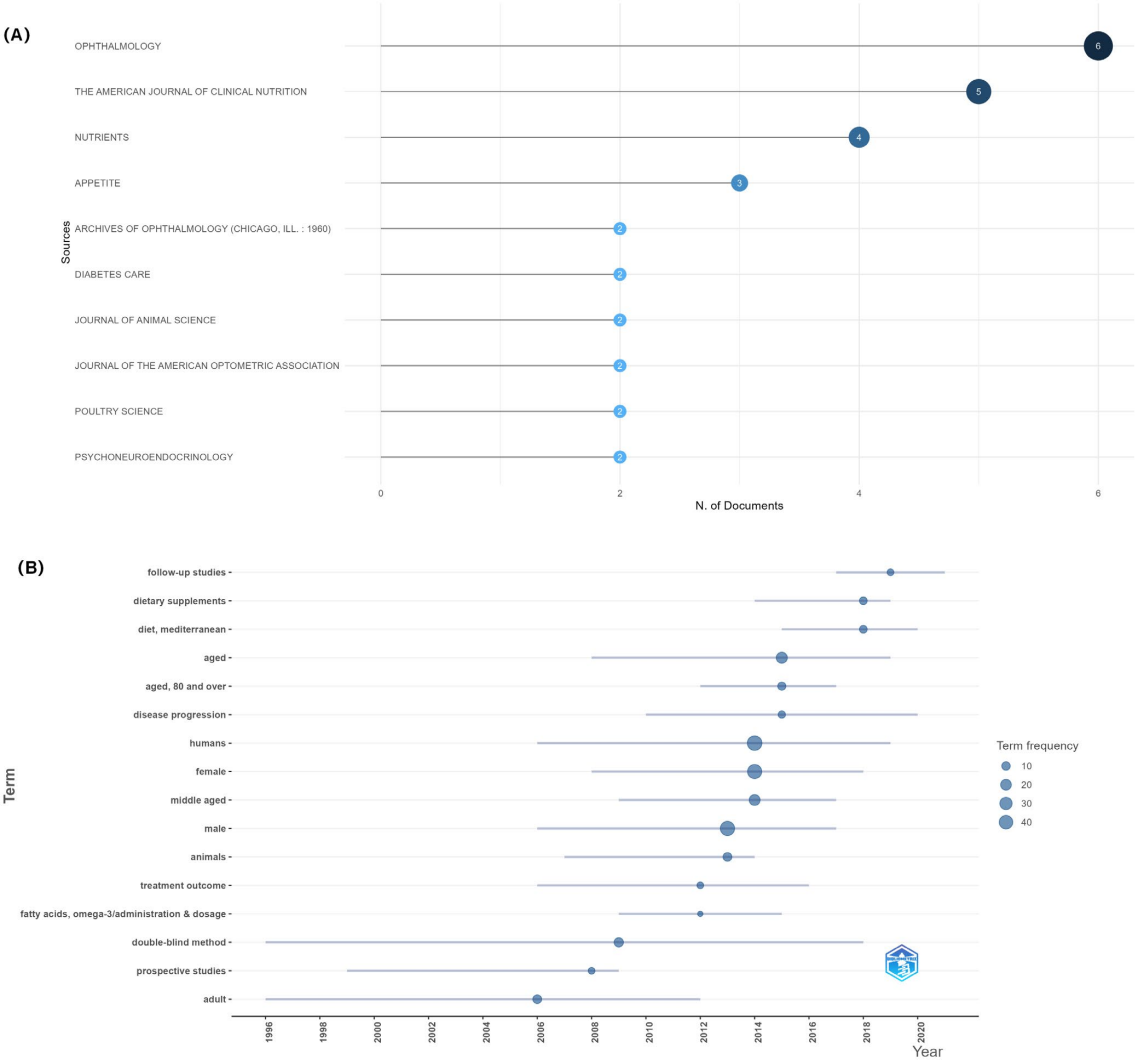
Bibliometric analysis of clinical trials on dietary patterns and eye diseases based on PubMed. (A) Top 10 journals publishing clinical trials on diet and eye health (B) Thematic evolution of MeSH terms over time.

Among the included clinical trial publications on diet and eye diseases, the most frequently represented journals were *Ophthalmology*, *The American Journal of Clinical Nutrition*, and *Nutrients*, indicating that this research area has garnered attention in both ophthalmology and nutritional science (Figure 10A). The temporal analysis of MeSH terms showed that early studies primarily focused on omega‐3 fatty acid intake, prospective study design, and double‐blind methods. Since 2014, however, there has been a notable rise in terms such as “Mediterranean diet”, “dietary supplements” and “follow‐up studies” (Figure 10B).

**FIGURE 11:**
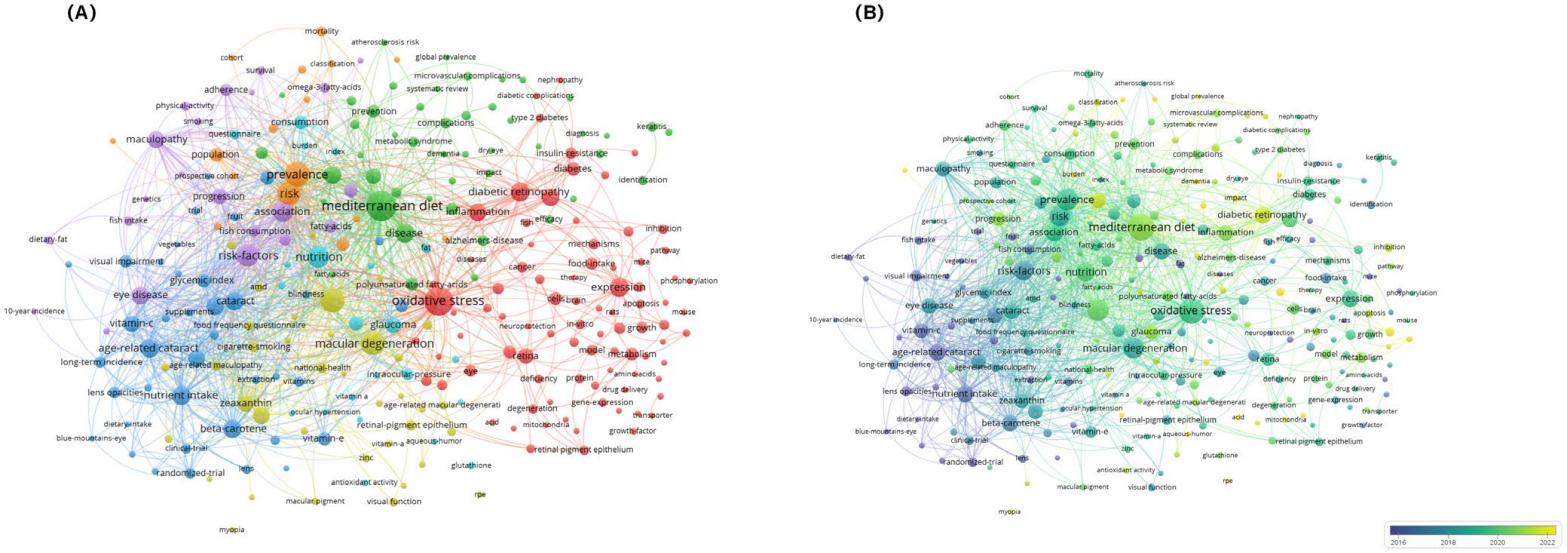
Keyword co‐occurrence and temporal trend mapping of researches on dietary patterns and eye diseases. (A) Co‐occurrence network of MeSH keywords. (B) Temporal overlay visualization.

Keyword co‐occurrence analysis revealed a diverse thematic structure within research on diet and eye diseases (Figure 11A). The major clusters included a diet–chronic disease theme characterized by terms such as “Mediterranean diet,” “metabolic syndrome,” and “disease prevention”; an antioxidant–degenerative eye disease theme centered around keywords like “cataract,” “vitamin C,” and “beta‐carotene”; and a molecular mechanism theme focusing on “oxidative stress,” “expression,” and “retina.” Additionally, the temporal analysis of keyword occurrence indicated that terms such as “Mediterranean diet,” “diabetic retinopathy,” and “gene expression” have emerged as recent research hotspots, whereas earlier focal points like “vitamin C” and “cataract” have shown a relative decline in attention (Figure 11B).

## 4 Discussion

In this nationally representative study of U.S. adults, we found that higher HEI‐2020 and DASH scores were significantly associated with a lower risk of retinopathy, composite outcome including cataract surgery, and any objectively eye disease. A higher MED score was specifically associated with a lower risk of retinopathy. Additionally, a nonlinear relationship was observed between HEI‐2020 and the risk of cataract. Subgroup analyses revealed substantial effect modification by population characteristics. The positive association between DII and cataract was significant among older adults, underweight individuals, alcohol drinkers, and those with hyperlipidemia. Conversely, higher HEI‐2020, MED, and DASH scores demonstrated stronger protective effects in individuals with hyperlipidemia, older adults, and non‐drinkers. The primary contributors to the protective effect of HEI‐2020 on eye diseases were whole grains, total vegetables, and refined grains. For DASH, whole grains emerged as the main contributor, while for the MED score, the nuts component played a predominant role.

This study found that adherence to the MED was associated with a lower risk of retinopathy, which is consistent with previous observational studies. For instance, a systematic review reported that higher intake of fruits, vegetables, dietary fiber, and fish, as well as overall adherence to the MED diet, was significantly associated with a reduced risk of diabetic retinopathy (Shah et al. 2022). A post hoc analysis of the PREDIMED randomized controlled trial also showed that among patients with type 2 diabetes, adherence to the MED dietary pattern was associated with an approximately 44% reduction in the risk of developing diabetic retinopathy (Salas‐Salvadó et al. 2019). We found that nuts may be a key protective component of the MED against retinopathy. This may be due to their richness in vitamin E, a fat‐soluble antioxidant that effectively neutralizes free radicals and prevents lipid peroxidation damage to photoreceptors and vascular endothelial cells in the retina (Hernández‐Olivas et al. 2022; Chandrinos et al. 2023). In addition, nuts are abundant in unsaturated fatty acids, particularly polyunsaturated fatty acids and alpha‐linolenic acid (Maguire et al. 2004). These fatty acids exert anti‐inflammatory and vascular protective effects, improve retinal microcirculation, and reduce chronic inflammation, which is a key pathological basis of retinopathy (Yang et al. 2023; Saccà et al. 2018).

Our data indicate a non‐linear association between HEI‐2020 scores and cataract risk, suggesting a potential threshold effect in how different levels of adherence to the HEI‐2020 diet influence lens health. This non‐linear trend may be related to a cumulative threshold of antioxidant load. The HEI‐2020 emphasizes the intake of antioxidant‐rich foods such as fruits, vegetables, whole grains, and plant‐based proteins, which contribute to reducing oxidative stress (Shams‐White et al. 2023). It is possible that low‐to‐moderate adherence does not provide sufficient antioxidant capacity to offset cumulative oxidative damage to lens proteins. Conversely, excessively high intakes of certain components without overall balance, or high energy intake from other sources, may attenuate the protective effect (Zhang, Qin, et al. 2024). Similar non‐linear relationships between dietary patterns and health outcomes have been reported, where inappropriate intake levels can be ineffective or even harmful (Zuo et al. 2024; Wang et al. 2021).

Another key finding is the inverse association between HEI‐2020 and DASH dietary scores with retinopathy, composite outcome including cataract surgery, and objectively measured eye disease. Although these scores are based on the U.S. Dietary Guidelines and have been widely used to assess overall diet quality, their predictive value for a broader spectrum of eye diseases has remained unclear. Our study suggests that high‐quality dietary patterns may not only help prevent metabolic diseases but also offer substantial protective benefits for eye health. Mechanistically, both HEI‐2020 and DASH emphasize intake of fiber‐, vitamin‐, mineral‐, and phytochemical‐rich foods such as fruits, vegetables, and whole grains, while limiting added sugars, saturated fats, and sodium (Shams‐White et al. 2023; Huang et al. 2024; Sacks et al. 2001). These dietary patterns may exert ocular protective effects through multiple mechanisms: by reducing systemic and ocular inflammation, mitigating oxidative stress, and protecting retinal neurons and macular structures (Bergandi et al. 2024); and by improving glycemic and lipid control, thereby lowering the risk of microvascular‐related eye conditions such as diabetic retinopathy (Shams‐White et al. 2023; Lari et al. 2021). Moreover, nutrients such as vitamins A, C, and potassium may enhance vascular function and retinal perfusion, strengthening the retina's resistance to pathological damage (Edo et al. 2022). An unexpected positive association between higher diet‐quality scores and cataract in the minimally adjusted model may reflect confounding and outcome‐specific ascertainment. In this study, cataract was proxied by self‐reported cataract surgery, an indicator of clinically treated cataract rather than total cataract burden. Cataract surgery uptake can be influenced by healthcare access, socioeconomic resources, and health‐seeking behaviors. Individuals with healthier dietary patterns often cluster with other favorable behaviors and greater preventive care utilization, which may increase the likelihood of receiving cataract surgery and thereby produce an apparent positive association in minimally adjusted analyses. These associations attenuated after adjusting for socioeconomic and cardiometabolic factors, supporting confounding rather than a harmful effect of healthy diets.

Our component contribution analysis further revealed that the protective effects of HEI‐2020 against eye diseases were mainly driven by whole grains, total vegetables, and refined grains. In the DASH pattern, whole grains emerged as the most protective component. The consistent contribution of whole grains in both scoring systems aligns with previous studies showing that whole grains, rich in dietary fiber, B vitamins, vitamin E, magnesium, and phytochemicals, may reduce oxidative stress, improve insulin sensitivity, and modulate glycemic and lipid levels (Aune et al. 2016; Gianotti et al. 2011), thereby interfering with the development of eye diseases through multiple pathways. Clinical studies have shown that high‐glycemic‐index (GI) and high‐glycemic‐load (GL) diets are significantly associated with cataracts, AMD, and diabetic retinopathy (Bejarano et al. 2024), whereas whole grains, having low GI/GL, may effectively reduce advanced glycation end products (AGEs) and delay ocular aging (Sanders et al. 2023). Total vegetable intake also played a key role in the HEI‐2020 score. Vegetables are major sources of antioxidant nutrients, including vitamin C, carotenoids (e.g., lutein and zeaxanthin), and polyphenols. Notably, lutein and zeaxanthin selectively accumulate in the macula, enhance protection against photo‐oxidative damage, and are essential in preventing ocular disease (Johra et al. 2020). Interestingly, refined grains also contributed significantly to HEI‐2020, suggesting that reducing refined grain intake is equally associated with lower eye disease risk. Refined grains are often over‐processed, stripped of nutrients, and rich in high‐GI carbohydrates, which may promote blood glucose fluctuations, inflammatory responses, and AGEs production (Sanders et al. 2023; Karl et al. 2017), thereby accelerating aging in the lens and retina. This further supports the recommendation to increase whole grain consumption.

Our subgroup analysis further highlighted how demographic and systemic factors modify the associations between diet and eye diseases. The positive association between the DII and cataract was more pronounced in individuals aged ≥ 60, those with BMI < 25, alcohol drinkers, and those with hyperlipidemia. This suggests that in populations with higher oxidative stress (e.g., older adults, those with dyslipidemia) or poor nutritional status (e.g., low BMI), pro‐inflammatory diets may exert more damaging effects on the lens (Vergroesen et al. 2023). The positive association between DII and retinopathy was particularly evident among alcohol drinkers, but reversed among non‐drinkers, indicating that alcohol intake may amplify the retinal damage induced by a pro‐inflammatory diet possibly through synergistic oxidative stress mechanisms (Sancho‐Tello et al. 2008). In hyperlipidemic individuals, DII was also positively associated with AMD, composite outcome including cataract surgery, and objectively diagnosed eye disease, supporting the notion that ocular tissues are more sensitive to dietary influences under chronic inflammatory conditions. In the HEI‐2020 pattern, an inverse association with glaucoma was observed only among non‐drinkers, suggesting that alcohol consumption may attenuate the neuroprotective effects of a healthy diet. The inverse association between HEI‐2020 and AMD was most pronounced in individuals with hyperlipidemia, in contrast to the DII results, further indicating that anti‐inflammatory and antioxidant dietary patterns such as HEI‐2020 may be particularly beneficial in populations with high inflammatory burden.

Additionally, the association between HEI‐2020 and retinopathy was modified by PIR and BMI, and in the hyperlipidemia subgroup, HEI‐2020 was significantly associated with reduced risk of composite outcome including cataract surgery and objectively diagnosed eye disease. These findings highlight the potential of HEI‐2020 for primary prevention of eye diseases in individuals with metabolic abnormalities, and emphasize the need to address nutritional vulnerabilities in low‐income and low‐BMI populations. For the MED, its inverse associations with glaucoma and AMD were mainly observed in older adults and non‐drinkers, indicating that plant‐rich, unsaturated fat‐based dietary patterns may be especially beneficial for retinal health, particularly under low‐inflammatory conditions. Furthermore, hyperlipidemia, educational attainment, and income levels significantly modified the associations between MED and retinopathy or other eye diseases. This aligns with the MED diet's mechanisms in improving lipid profiles and reducing oxidative stress and suggests that nutrition interventions may yield different effects in populations with lower health literacy. For the DASH dietary pattern, its protective associations with glaucoma, AMD, retinopathy, and overall eye disease were more evident in individuals with hyperlipidemia, older adults, women, and alcohol drinkers. PIR also showed interaction effects with objectively diagnosed eye disease under the DASH pattern, suggesting that DASH may be particularly effective in enhancing microvascular health and reducing inflammation in high‐risk or physiologically stressed populations. Notably, in individuals aged ≥ 60, DASH was significantly associated with reduced risk of objectively diagnosed eye disease, indicating its potential as an intervention for delaying age‐related ocular disorders.

The relationship between diet and eye diseases can be explained through several key physiological mechanisms. First, the anti‐inflammatory and antioxidant properties of healthy dietary patterns are crucial. These diets are rich in antioxidants, fiber, vitamins, and minerals, which help reduce systemic inflammation and oxidative stress, both of which are key pathological factors in retinal damage (Liu, Chen, Ning, et al. 2025). Specifically, the Mediterranean and DASH diets, which are rich in fruits, vegetables, nuts, and whole grains, help reduce inflammation and enhance antioxidant capacity, thereby alleviating retinal damage. Second, the connection between cardiovascular health and eye diseases cannot be overlooked. Cardiovascular disease (CVD) is a significant factor influencing retinal health, especially for individuals with diabetes and hypertension (Liu, Chen, Hu, and Zhu 2025; Zheng et al. 2025). The Mediterranean and DASH diets improve cardiovascular health by regulating blood lipids and glucose, thereby reducing the risk of eye diseases related to microvascular damage, such as diabetic retinopathy. Healthy dietary patterns help maintain vascular health, reducing the occurrence of microvascular diseases, which is crucial for blood circulation in the eyes and retinal health. Furthermore, while this study did not directly explore the relationship between the gut microbiome and eye diseases, existing research suggests that gut health has a profound impact on overall health, particularly in the development and progression of chronic diseases (Zysset‐Burri et al. 2023). Gut dysbiosis can lead to systemic inflammation, which in turn exacerbates conditions like diabetes and cardiovascular diseases, both of which are closely linked to eye diseases (Clemente et al. 2018). Diets can influence the gut microbiome, thereby indirectly affecting systemic inflammation and metabolic status, which in turn affects the risk of eye diseases.

The strength of this study lies in its use of NHANES, which provide a nationally representative sample with rigorous quality control, enabling a more accurate reflection of dietary behaviors and their associations with eye disease risks in U.S. adults compared to small‐sample or single‐center studies. In terms of clinical value, the study evaluated the relationships between multiple dietary patterns and major eye diseases from a holistic perspective, while also conducting subgroup interaction analyses to reveal how demographic and metabolic factors modify these associations. Additionally, by identifying protective dietary components, the findings have clear implications for daily dietary guidance, community nutrition education, integrated chronic disease prevention, and dietary counseling in ophthalmology clinics, thus emphasizing the critical role of nutrition in eye disease prevention. Complementing these findings, our bibliometric analysis further supports the growing scientific interest in the role of healthy dietary patterns, particularly the Mediterranean diet in ocular health. Keyword clustering revealed consistent themes with our empirical results, including diet–metabolic interactions, antioxidant‐based interventions, and molecular mechanisms such as oxidative stress and gene expression. The alignment between real‐world data and research trends underscores the potential of anti‐inflammatory and nutrient‐rich diets in eye disease prevention.

However, several limitations warrant consideration. First, the cross‐sectional design limits causal inference. Longitudinal studies are needed to confirm the temporal sequence of diet and eye disease development. Second, dietary intake was self‐reported and subject to recall bias, although the use of two 24‐h recalls and validated scoring algorithms helps to mitigate this issue. Third, cataract was defined using self‐reported cataract surgery, which captures primarily clinically significant, treated cataract and may underestimate the burden of untreated or undiagnosed cataract. Future studies should incorporate objective lens assessments (e.g., slit‐lamp grading or standardized photographic grading) to better capture the full spectrum of cataract. Fourth, potential selection bias and limited generalizability should be acknowledged. Objectively assessed retinal outcomes (retinopathy, AMD, and glaucoma) were available only among eligible participants who attended the MEC examination and had gradable fundus photographs; therefore, the analytic sample may differ systematically from excluded participants, which could affect the representativeness of the estimates. Finally, residual confounding cannot be ruled out, particularly from unmeasured factors such as medication use and physical activity, which were unavailable in our dataset. While we adjusted for major cardiometabolic comorbidities (diabetes, hypertension, hyperlipidemia) that are closely linked to these factors, future studies with comprehensive medication and physical activity data are needed to further refine confounding control.

## 5 Conclusion

This study found that higher HEI‐2020, DASH, and MED dietary scores were significantly associated with a lower risk of retinopathy, and that HEI‐2020 and DASH were also inversely associated with the overall risk of eye diseases. A non‐linear association was observed between MED adherence and cataract risk, suggesting a potential threshold effect. These associations varied across different population subgroups. Complementary bibliometric analysis further revealed a growing body of literature supporting the role of healthy diets, especially the Mediterranean diet, in ocular health. The alignment between our empirical findings and bibliometric trends underscores a growing scientific consensus and reinforces the call for high‐quality prospective studies and clinical trials to validate the preventive potential of dietary interventions.

## Author Contributions

The study's conception and design were meticulously crafted by Kunhong Xiao, and Jiajia Gao. Kunhong Xiao and Li Li undertook the drafting of the manuscript, with critical intellectual input from all authors during its revision. Statistical analysis was diligently performed by Kunhong Xiao and Ruiye Chen Administrative, technical, and material support were provided by Yiyan Zhang, JiaJia Gao, Jiahao Liu, Chufeng Gu, and Li Li The overall study was overseen by Yan Huang, Jiahao Liu and Li Li ensuring its successful execution and completion.

## Funding

The work was supported by the Natural Science Foundation of Fujian Province, China (grant number: 2024J011019) to L.L., the Fujian Provincial Health System Young and Middle‐Aged Key Talent Development Program (grant number: 2025GGA002) to L.L., and the Fujian Medical University Qihang Foundation (grant number: 2020QH1160) to J.G.

## Ethics Statement

The NHANES protocol was approved by the National Center for Health Statistics (NCHS) Research Ethics Review Board and was conducted in accordance with the principles of the Declaration of Helsinki. Written informed consent was obtained from all participants. This study involved secondary analysis of anonymized, publicly available data and did not require further ethical approval.

## Conflicts of Interest

The authors declare no conflicts of interest.

## Supporting information


**Table S1:** Overview of dietary pattern scores and their component‐based scoring criteria.
**Table S2:** Baseline Characteristics of participants with and without composite outcome including cataract surgery and composite outcome excluding cataract surgery.
**Table S3:** Sensitivity analysis of dietary patterns and retinopathy severity using survey‐weighted logistic regression.
**Figure S1:** Dose response relationships between dietary indices and the risk of composite outcome including cataract surgery and composite outcome excluding cataract surgery in NHANES 2005–2008. (A) DII and composite outcome including cataract surgery, (B) DII and composite outcome excluding cataract surgery, (C) HEI‐2020 and composite outcome including cataract surgery, (D) HEI‐2020 and composite outcome excluding cataract surgery, (E) MED and composite outcome including cataract surgery, (F) MED and composite outcome excluding cataract surgery, (G) DASH and composite outcome including cataract surgery, (H) DASH and composite outcome excluding cataract surgery.
**Figure S2:** Stratified analyses of the associations between dietary indices and composite outcome including cataract surgery in NHANES 2005–2008. (A) DII and composite outcome including cataract surgery, (B) HEI 2020 and composite outcome including cataract surgery, (C) MED and composite outcome including cataract surgery, (D) DASH and composite outcome including cataract surgery.
**Figure S3:** Stratified analyses of the associations between dietary indices and composite outcome excluding cataract surgery in NHANES 2005–2008. (A) DII and composite outcome excluding cataract surgery, (B) HEI 2020 and composite outcome excluding cataract surgery, (C) MED and composite outcome excluding cataract surgery, (D) DASH and composite outcome excluding cataract surgery.

## Data Availability

All data analyzed in this study can be accessed in https://wwwn.cdc.gov/nchs/nhanes/Default.aspx.
