# Supplementary material for: Healthy Dietary Patterns and Risk of Major Eye Diseases: Evidence From Nationally Population‐Based Data and Bibliometric Analysis
Source: Food Sci Nutr. 2026 Mar 3;14(3):e71528. doi: 10.1002/fsn3.71528 (PMC12954465; doi:10.1002/fsn3.71528)
Supplement: Supplementary file 1 — Table S1: Overview of dietary pattern scores and their component‐based scoring criteria. Table S2: Baseline Characteristics of participants with and without composite outcome including cataract surgery and composite outcome excluding cataract surgery. Table S3: Sensitivity analysis of dietary patterns and retinopathy severity using survey‐weighted logistic regression. Figure S1: Dose response relationships between dietary indices and the risk of composite outcome including cataract surgery and composite outcome excluding cataract surgery in NHANES 2005–2008. (A) DII and composite outcome including cataract surgery, (B) DII and composite outcome excluding cataract surgery, (C) HEI‐2020 and composite outcome including cataract surgery, (D) HEI‐2020 and composite outcome excluding cataract surgery, (E) MED and composite outcome including cataract surgery, (F) MED and composite outcome excluding cataract surgery, (G) DASH and composite outcome including cataract surgery, (H) DASH and composite outcome excluding cataract surgery. Figure S2: Stratified analyses of the associations between dietary indices and composite outcome including cataract surgery in NHANES 2005–2008. (A) DII and composite outcome including cataract surgery, (B) HEI 2020 and composite outcome including cataract surgery, (C) MED and composite outcome including cataract surgery, (D) DASH and composite outcome including cataract surgery. Figure S3: Stratified analyses of the associations between dietary indices and composite outcome excluding cataract surgery in NHANES 2005–2008. (A) DII and composite outcome excluding cataract surgery, (B) HEI 2020 and composite outcome excluding cataract surgery, (C) MED and composite outcome excluding cataract surgery, (D) DASH and composite outcome excluding cataract surgery. [file FSN3-14-e71528-s001.docx]

**Supplementary materials**

**Supplementary Table 1. Overview of dietary pattern scores and their component-based scoring criteria.**

| Dietary Index | Score range/Components | Definition in DietaryIndex |
| --- | --- | --- |
| HEI-2020 | 0–100 points; 13 components (8 adequacy, 5 moderation) | Uses both Day 1 and Day 2 24h recalls to derive food group/nutrient intakes from NHANES FPED and nutrient files; scores components proportionally based on per 1000 kcal standards (e.g., ≥0.8 cup fruit/1000 kcal = 5 points; ≤1.8 oz refined grains/1000 kcal = 10 points; Added sugars ≤6.5% energy). |
| Mediterranean diet score | 0–9 points; 9 components | For each component, assigns 1 point if intake is above (or below for meat/dairy) the sex-specific median; moderate alcohol gets 1 point. Component include vegetables, legumes, fruit/nuts, cereals (whole grains), fish, MUFA+PUFA:SFA ratio, meat & meat products, dairy products, alcohol. |
| DASH score | 8–40 points; 8 components (fruits, vegetables, nuts/legumes, whole grains, low-fat dairy, sodium, red/processed meats, sweetened beverages) | Rank participants into quintiles for each component; beneficial components scored 1–5 points from lowest to highest quintile; unhealthy components (sodium, red meat/processed meats, sweet drinks) reverse-scored. |
| DII Index | Theoretical range ~ −8.87 to +7.98 (actual ~ −5.5 to +5.5); incorporates up to 45 food parameters | For each nutrient/food parameter, computes a z-score relative to a global reference, converts to a centered percentile and multiplies by an inflammatory effect score; sums across parameters to give a pro- or anti-inflammatory score. |

**Supplementary Table 2. Baseline Characteristics of Participants With and Without composite outcome including cataract surgery and composite outcome excluding cataract surgery**

| **Characteristic** | **Overall**  N = 4241 | **Non-**  **composite outcome including cataract surgery**  N = 3067 | **composite outcome including cataract surgery**  N = 1174 | **p-value** | **Non-**  **composite outcome excluding cataract surgery**  N = 3361 | **composite outcome excluding cataract surgery**  N = 880 | **p-value** |
| --- | --- | --- | --- | --- | --- | --- | --- |
| **Age** | 54.00 (47.00, 64.00) | 52.00 (46.00, 60.00) | 66.00 (54.00, 76.00) | <0.001 | 53.00 (46.00, 62.00) | 63.00 (52.00, 74.00) | <0.001 |
| **Sex** |  |  |  | 0.018 |  |  | <0.001 |
| Female | 2141 (53%) | 1585 (54%) | 556 (51%) |  | 1750 (54%) | 391 (46%) |  |
| Male | 2100 (47%) | 1482 (46%) | 618 (49%) |  | 1611 (46%) | 489 (54%) |  |
| **Race** |  |  |  | 0.2 |  |  | 0.015 |
| Non-Hispanic White | 2411 (80%) | 1710 (80%) | 701 (80%) |  | 1923 (80%) | 488 (78%) |  |
| Non-Hispanic Black | 802 (8.5%) | 570 (8.2%) | 232 (9.5%) |  | 604 (8.0%) | 198 (11%) |  |
| Mexican American | 633 (5.1%) | 482 (5.2%) | 151 (4.7%) |  | 508 (5.0%) | 125 (5.3%) |  |
| Other Hispanic | 275 (2.9%) | 209 (2.9%) | 66 (2.9%) |  | 224 (2.9%) | 51 (3.3%) |  |
| Other Race | 120 (3.8%) | 96 (4.2%) | 24 (2.6%) |  | 102 (4.1%) | 18 (2.4%) |  |
| PIR |  |  |  | >0.9 |  |  | 0.3 |
| At or above poverty line | 3633 (92%) | 2625 (92%) | 1008 (92%) |  | 2888 (92%) | 745 (91%) |  |
| Below poverty line | 608 (8.1%) | 442 (8.1%) | 166 (8.1%) |  | 473 (7.9%) | 135 (9.1%) |  |
| **Education** |  |  |  | <0.001 |  |  | <0.001 |
| Some College or AA degree | 1111 (29%) | 822 (29%) | 289 (28%) |  | 888 (29%) | 223 (28%) |  |
| High School Grad/GED | 1051 (26%) | 747 (25%) | 304 (29%) |  | 821 (25%) | 230 (29%) |  |
| College Graduate or above | 935 (29%) | 738 (31%) | 197 (21%) |  | 796 (30%) | 139 (20%) |  |
| 9-11th Grade | 631 (11%) | 439 (10.0%) | 192 (13%) |  | 484 (10%) | 147 (13%) |  |
| Less Than 9th Grade | 513 (5.9%) | 321 (5.0%) | 192 (9.1%) |  | 372 (5.2%) | 141 (9.1%) |  |
| BMI | 28.19 (24.75, 32.41) | 28.15 (24.69, 32.43) | 28.31 (24.89, 32.28) | 0.9 | 28.12 (24.60, 32.31) | 28.58 (25.14, 32.58) | 0.2 |
| **Glycohemoglobin (%)** | 5.50 (5.20, 5.80) | 5.40 (5.20, 5.70) | 5.70 (5.40, 6.10) | <0.001 | 5.50 (5.20, 5.70) | 5.70 (5.40, 6.20) | <0.001 |
| **Drink status** |  |  |  | <0.001 |  |  | <0.001 |
| Drink | 2930 (73%) | 2181 (75%) | 749 (66%) |  | 2364 (74%) | 566 (67%) |  |
| Non drink | 1311 (27%) | 886 (25%) | 425 (34%) |  | 997 (26%) | 314 (33%) |  |
| **Diabetes Status** |  |  |  | <0.001 |  |  | <0.001 |
| No diabetes | 3613 (89%) | 2751 (93%) | 862 (78%) |  | 2998 (92%) | 615 (75%) |  |
| Diabetes | 628 (11%) | 316 (7.5%) | 312 (22%) |  | 363 (7.9%) | 265 (25%) |  |
| **Smoking Status** |  |  |  | <0.001 |  |  | 0.007 |
| Never Smoked | 2003 (48%) | 1484 (50%) | 519 (44%) |  | 1612 (49%) | 391 (43%) |  |
| Former Smoker | 1404 (32%) | 933 (30%) | 471 (39%) |  | 1064 (31%) | 340 (37%) |  |
| Current Smoker | 834 (20%) | 650 (20%) | 184 (18%) |  | 685 (20%) | 149 (19%) |  |
| **Hypertension Status** |  |  |  | <0.001 |  |  | <0.001 |
| No Hypertension | 2318 (59%) | 1829 (62%) | 489 (47%) |  | 1946 (61%) | 372 (48%) |  |
| Hypertension | 1923 (41%) | 1238 (38%) | 685 (53%) |  | 1415 (39%) | 508 (52%) |  |
| Hyperlipidemia Status |  |  |  | 0.10 |  |  | 0.11 |
| No Hyperlipidemia | 3513 (82%) | 2517 (82%) | 996 (85%) |  | 2766 (82%) | 747 (85%) |  |
| Hyperlipidemia | 728 (18%) | 550 (18%) | 178 (15%) |  | 595 (18%) | 133 (15%) |  |
| DII score | 2.60 (1.12, 3.95) | 2.53 (1.08, 3.85) | 2.83 (1.26, 4.25) | 0.021 | 2.56 (1.09, 3.90) | 2.79 (1.16, 4.15) | 0.14 |
| HEI2020 score | 51.37 (43.70, 60.05) | 51.16 (43.43, 59.78) | 52.20 (44.51, 61.25) | 0.035 | 51.31 (43.57, 59.94) | 51.65 (44.20, 60.59) | 0.4 |
| MED score | 3.50 (2.50, 4.50) | 3.50 (2.50, 4.50) | 3.50 (3.00, 4.50) | 0.4 | 3.50 (2.50, 4.50) | 3.50 (2.50, 4.50) | 0.5 |
| DASH score | 23.00 (20.00, 27.00) | 23.00 (19.50, 27.00) | 23.50 (20.50, 27.00) | 0.2 | 23.00 (20.00, 27.00) | 23.00 (20.00, 26.50) | 0.5 |

Notes: Continuous variables were summarized using means and interquartile ranges, while categorical variables were presented as unweighted counts and weighted percentages. Comparisons between participants with and without major eye diseases were conducted using *t*-tests for continuous variables and design-adjusted Rao–Scott Pearson χ² tests for categorical variables. Bolded variables indicate statistically significant between-group differences.

**Supplementary Table 3.Sensitivity Analysis of Dietary Patterns and Retinopathy Severity Using Survey-Weighted Logistic Regression**

| **Exposure** | **OR (95% CI)** | **P value** |
| --- | --- | --- |
| DII_ALL | 0.956 (0.835–1.094) | 0.512 |
| HEI2020_ALL | 1.005 (0.982–1.030) | 0.658 |
| MED_ALL | 1.062 (0.856–1.318) | 0.583 |
| DASH_ALL | 1.049 (0.988–1.115) | 0.119 |


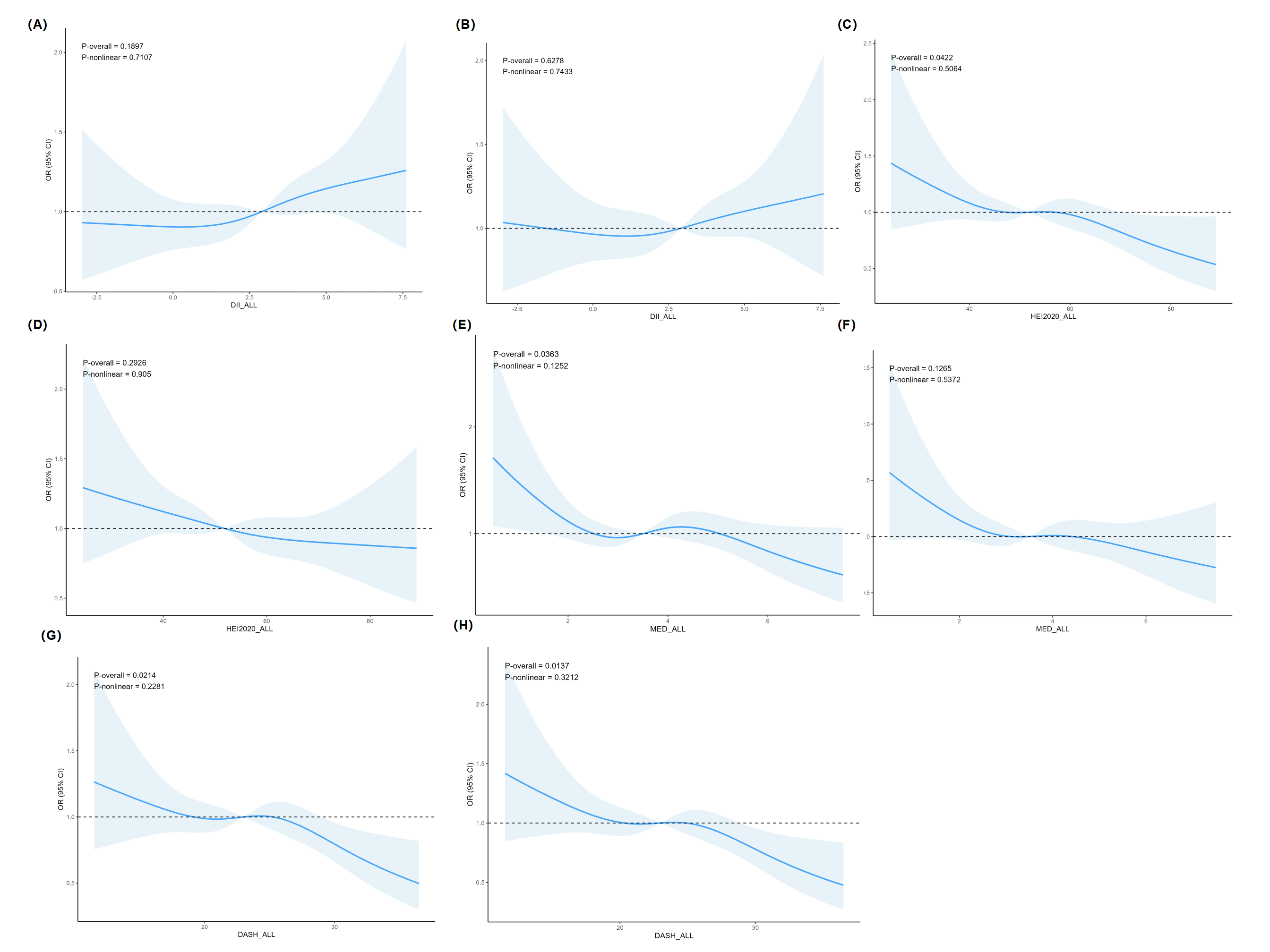


**Supplementary Figure 1.Dose response relationships between dietary indices and the risk of composite outcome including cataract surgery and composite outcome excluding cataract surgery in NHANES 2005–2008.** (A) DII and composite outcome including cataract surgery, (B) DII and composite outcome excluding cataract surgery, (C) HEI-2020 and composite outcome including cataract surgery, (D) HEI-2020 and composite outcome excluding cataract surgery, (E) MED and composite outcome including cataract surgery, (F)MED and composite outcome excluding cataract surgery, (G)DASH and composite outcome including cataract surgery, (H)DASH and composite outcome excluding cataract surgery.


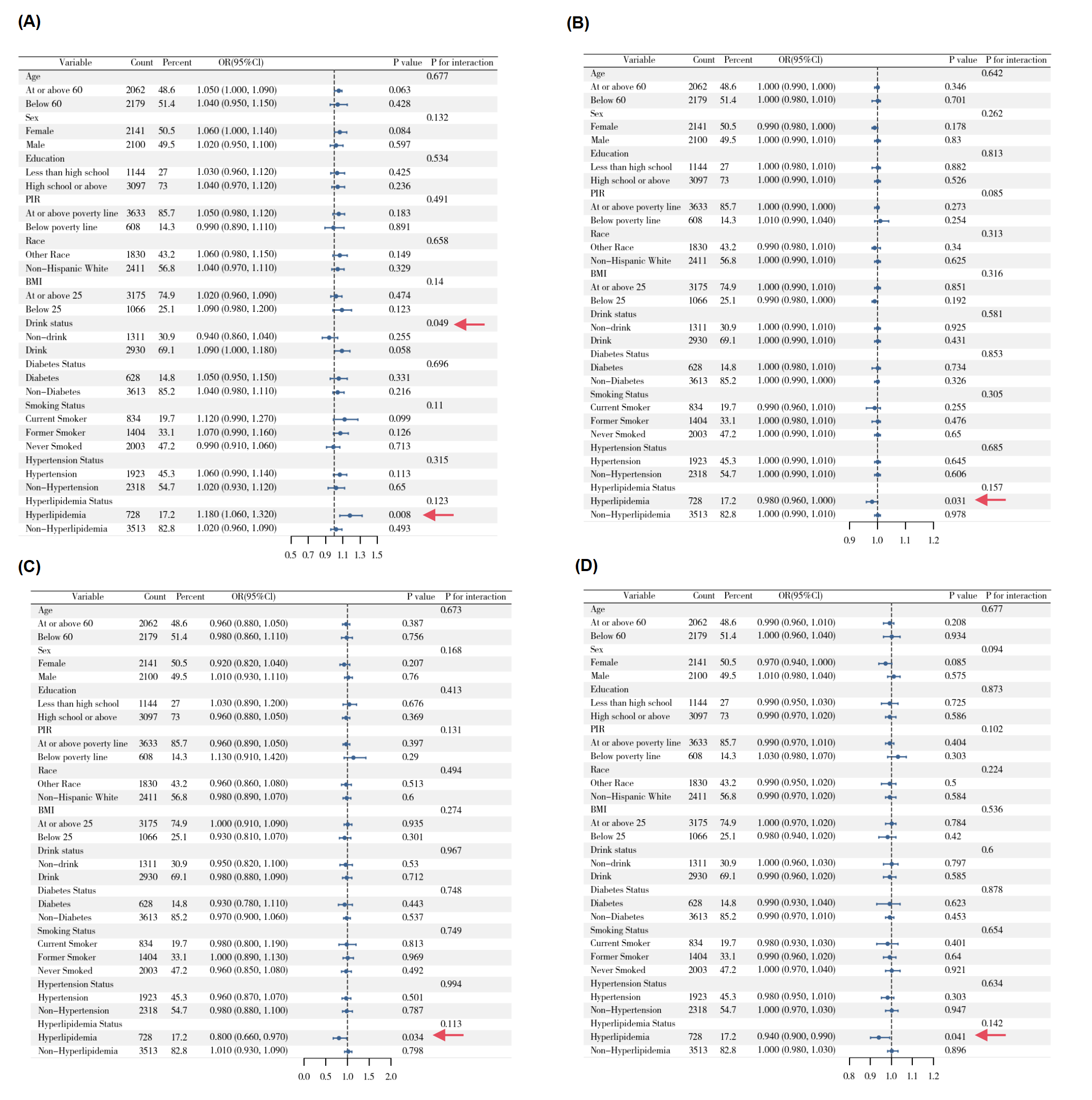


**Supplementary Figure 2. Stratified analyses of the associations between dietary indices and composite outcome including cataract surgery in NHANES 2005–2008.** (A)DII and composite outcome including cataract surgery, (B)HEI 2020 and composite outcome including cataract surgery, (C)MED and composite outcome including cataract surgery, (D)DASH and composite outcome including cataract surgery.


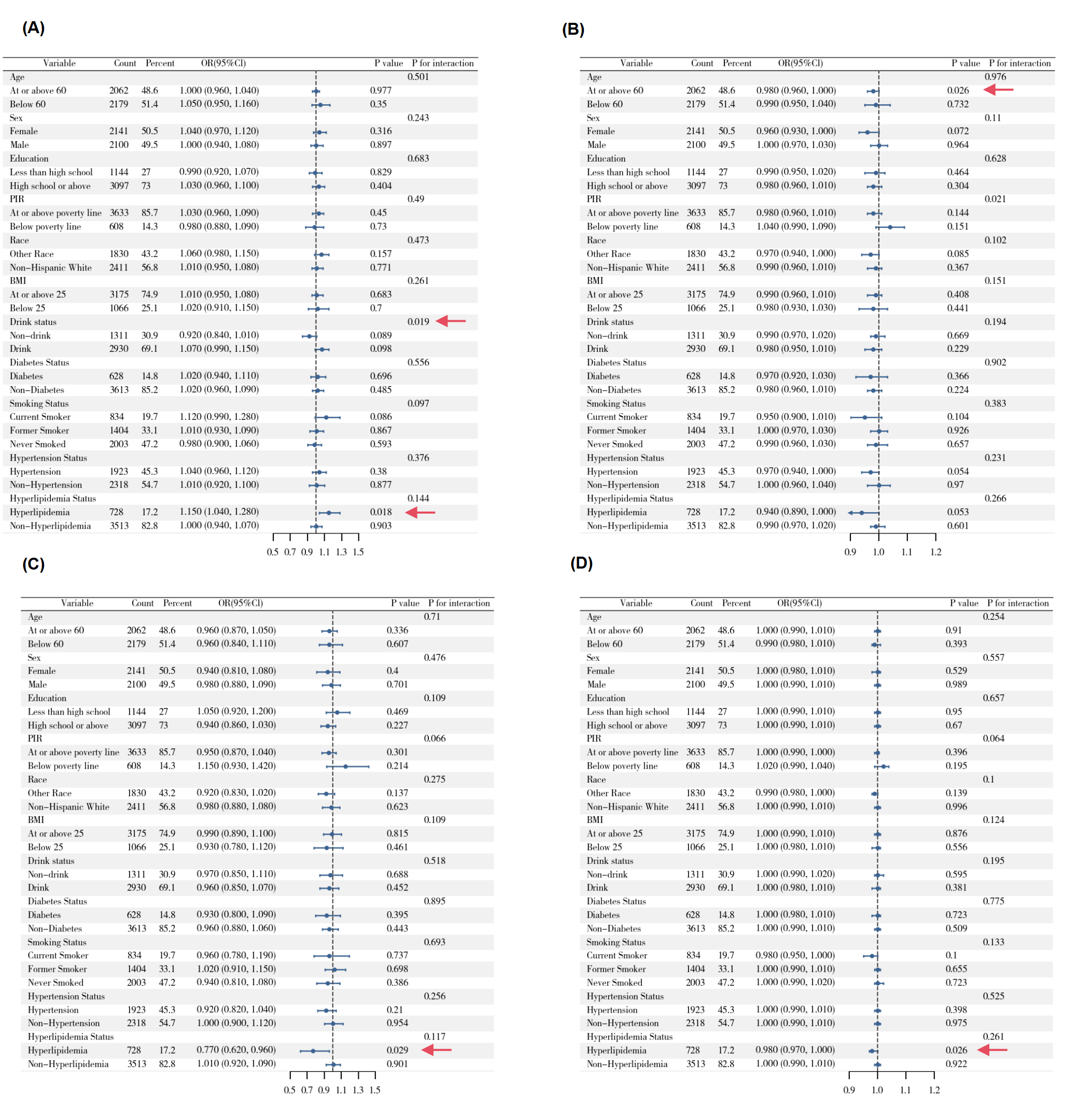


**Supplementary Figure 3.Stratified analyses of the associations between dietary indices and composite outcome excluding cataract surgery in NHANES 2005–2008.** (A)DII and composite outcome excluding cataract surgery, (B)HEI 2020 and composite outcome excluding cataract surgery, (C)MED and composite outcome excluding cataract surgery, (D)DASH and composite outcome excluding cataract surgery.
